# Supersonic turbulent flow simulation using a scalable parallel modal discontinuous Galerkin numerical method

**DOI:** 10.1038/s41598-019-50546-w

**Published:** 2019-10-08

**Authors:** Tomas Houba, Arnob Dasgupta, Shivasubramanian Gopalakrishnan, Ryan Gosse, Subrata Roy

**Affiliations:** 1 SurfPlasma Inc, Gainesville, FL 32601 USA; 2 Applied Physics Research Group, Department of Mechanical and Aerospace Engineering, University of Florida, Gainesville, FL 32601 USA; 3 Department of Mechanical Engineering, Indian Institute of Technology Bombay, Mumbai, 400076 India; 4 Vanilla Peak Ct, Vail, AZ 85641 USA

**Keywords:** Aerospace engineering, Computational science

## Abstract

The scalability and efficiency of numerical methods on parallel computer architectures is of prime importance as we march towards exascale computing. Classical methods like finite difference schemes and finite volume methods have inherent roadblocks in their mathematical construction to achieve good scalability. These methods are popularly used to solve the Navier-Stokes equations for fluid flow simulations. The discontinuous Galerkin family of methods for solving continuum partial differential equations has shown promise in realizing parallel efficiency and scalability when approaching petascale computations. In this paper an explicit modal discontinuous Galerkin (DG) method utilizing Implicit Large Eddy Simulation (ILES) is proposed for unsteady turbulent flow simulations involving the three-dimensional Navier-Stokes equations. A study of the method was performed for the Taylor-Green vortex case at a Reynolds number ranging from 100 to 1600. The polynomial order *P* = 2 (third order accurate) was found to closely match the Direct Navier-Stokes (DNS) results for all Reynolds numbers tested outside of Re = 1600, which had a normalized RMS error of 3.43 × 10^−4^ in the dissipation rate for a 60^3^ element mesh. The scalability and performance study of the method was then conducted for a Reynolds number of 1600 for polynomials orders from *P* = 2 to *P* = 6. The highest order polynomial that was tested (*P* = 6) was found to have the most efficient scalability using both the MPI and OpenMP implementations.

## Introduction

Advances in modern computer hardware have enabled numerical computations to reach progressively larger scales. To handle the challenging and costly simulations, parallel computations have become widespread in both research and production Computational Fluid Dynamics (CFD) and other Computer-Aided Engineering (CAE) codes. To keep up with the demand for increasingly larger and more complex numerical models, the scalability and efficiency of a parallel implementation for a numerical discretization is an important factor. One way to improve the parallel efficiency of CFD software is to optimize the underlying code. Examples of these types of optimizations are the improvement of loop-level parallelism, serial efficiency of the code^1^, reducing the number of cache misses and optimizing the achievable memory bandwidth^2^.

Another possible path to improve the parallel efficiency is to consider the numerical method implemented in the software. One promising direction is the application of high-order methods for massively parallel CFD. In the CFD community, high-order methods are considered to be those which are third order and higher^3^. Low-order schemes are widely used in CFD, but there are applications for which they are considered insufficient, including turbulence, aeroacoustics, boundary layer flows, vortical flows, shock-boundary layer interactions and others^4^. For these types of flows, low-order methods require extremely small discretization scale lengths to accurately resolve the unsteady vortices over relevant length and time scales. This has led to a large amount of research in high-order methods aimed at solving physics problems which are not well suited to low-order methods. Outside of this “physics” argument for use of high-order methods, there is the issue of parallel scalability.

Since higher order polynomial approximations require more calculations to be carried out per element, it is expected that the scheme will exhibit a higher efficiency when higher order polynomials are used. The parallel algorithm requires a finite setup and communication time, which decreases its efficiency below the ideal linear speed-up. This overhead time depends on the scale of the parallel simulation, i.e. the number of parallel tasks or threads used. Since the higher order polynomials spend a longer time calculating the solution on a per degree of freedom basis, it is expected that the overhead time would be more negligible in comparison.

The objective of this paper is to demonstrate a scalable, parallel, high-order description of modal Discontinuous Galerkin (DG) elements for supersonic, turbulent boundary layer flows using Runge-Kutta explicit time marching. The spatial discretization scheme considered in the DG method can be made high-order by increasing the approximation order *P* of the interpolating polynomial. Polynomial approximations ranging from *P* = 2 to *P* = 6 are compared for a canonical problem of isotropic turbulence to study their parallel efficiency. In addition, the computational cost required to reach the same error as a lower-order polynomial is considered. This is an important metric to obtain the full picture of the computational cost of the different polynomial orders. Other authors have proposed using operation count instead of runtime comparisons, and found that for implicit solvers, high-order methods were more efficient than low-order ones^5^. Parallel scalability is important, but only if the underlying serial computational cost is not prohibitively expensive to the point where the benefit gained from a better scalability is lost. In addition to the isotropic turbulence, the method was also validated on a zero-pressure gradient supersonic Mach 2.25 turbulent boundary layer flow over a flat plate.

This paper is organized as follows. The next section describes the governing equations used in the study. Then an overview of the numerical method, including the DG spatial discretization, the numerical fluxes and the time integration is given. The ensuing section provides the background and results for the isotropic turbulence (Taylor-Green vortex) test case. Following that the results of the parallel scalability studies and performance comparisons of different polynomial orders are presented. Then, the turbulent boundary layer flow solution for a supersonic flow over a flat plate is documented. Finally the conclusions from these studies are summarized.

## Governing Equations

To understand the fluid mechanics, one must appreciate the partial differential equations which govern fluid flow. This section describes these governing equations as well as other equations involved in this study.

### Compressible Navier-Stokes equations

For a compressible Newtonian fluid, the multi-dimensional N-S equations in normalized conservative form can be written as

1 $$\frac{\partial {\boldsymbol{\rho }}}{\partial {\boldsymbol{t}}}+\nabla \cdot ({\boldsymbol{\rho }}v)=0$$

2 $$\frac{{\rm{\partial }}({\boldsymbol{\rho }}v)}{{\rm{\partial }}{\boldsymbol{t}}}+{\rm{\nabla }}\cdot ({\boldsymbol{\rho }}v{{\boldsymbol{v}}}^{{\boldsymbol{\top }}}+p{\bf{I}}-\bar{{\boldsymbol{\tau }}})=0$$

3 $$\frac{\partial ({\boldsymbol{\rho }}E)}{\partial {\boldsymbol{t}}}+\nabla \cdot [({\boldsymbol{\rho }}E+p){\boldsymbol{v}}-k\nabla T-{\boldsymbol{v}}\cdot \bar{{\boldsymbol{\tau }}}]=0$$

4 $$\bar{{\boldsymbol{\tau }}}=[\begin{array}{ccc}{\tau }_{xx} & {\tau }_{xy} & {\tau }_{xz}\\ {\tau }_{yx} & {\tau }_{yy} & {\tau }_{yz}\\ {\tau }_{zx} & {\tau }_{zy} & {\tau }_{zz}\end{array}];{\tau }_{ij}=\mu (\frac{{\rm{\partial }}{v}_{i}}{{\rm{\partial }}{x}_{j}}+\frac{{\rm{\partial }}{v}_{j}}{{\rm{\partial }}{x}_{i}}-\frac{2}{3}\frac{{\rm{\partial }}{v}_{k}}{{\rm{\partial }}{x}_{k}}{\delta }_{ij});i,j,k=x,y,z$$

5 $$p=(\gamma -1)[\rho E-\frac{1}{2}\rho {|{\bf{v}}|}^{2}];T=\frac{p}{\rho R};R=\frac{1}{\gamma {M}^{2}};k=\frac{\mu {c}_{p}}{{\rm{\Pr }}}$$

Here $\bar{{\boldsymbol{\tau }}}$ denotes the viscous stress tensor which is given by Eq. (4). The term *μ* in the viscous stresses is the dynamic viscosity of the fluid and Sutherland’s law is used to define it. The term *k* denotes the thermal conductivity of the fluid with *T* being its temperature. This term comes from the Fourier’s Law of heat conduction. The thermal conductivity is obtained using the dynamic viscosity *μ*, Prandtl number (Pr) and specific heat (*c*_*p*_) of the fluid given by Eq. (5). The velocity vector is denoted by **v**, which includes the three components, *u*, *v* and *w* in streamwise (*x*), wall normal (*y*) and spanwise (*z*) directions, respectively.

## Numerical Method

DG finite element was first presented by Reed and Hill^6^ to solve the neutron transport equations. Due to its inherent advantage of solving linear equation systems on an element-by-element basis, it has become one of the most promising computational technique to solve large equation systems with high parallel efficiency, even allowing the numerical formulation to approach an “embarrassingly parallel problem”. However, the next challenge was to solve the nonlinear systems of equations such as the hyperbolic conservation laws, which are prominent in most physical systems. For this, an explicit version of this method was devised^7^ which employed the use of Runge – Kutta time discretization with a Total Variation Diminishing in the Means (TVDM) and Total Variation Bounded (TVB) slope limiter. This method was called the RKDG method. This was extended to high order RKDG methods^8^ which showed *P* + 1 order of convergence for *P* order space discretization.

The development of DG methods for nonlinear hyperbolic systems occurred rapidly over the past two decades. The improvement of the computer architecture (for example, the advent of petascale computing machines) combined with the need to solve both hyperbolic and elliptic problems led to the extension of this method to convection-diffusion type problems. The first study of this form of equations was conducted on hydrodynamic models for semiconductor device simulations^9,10^. This was further studied for compressible Navier Stokes equations^11^ to achieve higher order of accuracy. It involved the simple breakdown of the second order equation into two first order equations with *U* and *dU* as independent variables and then solving the system using the original RKDG method. This method also known as the first Bassi – Rebay (BR1) method^11^ was further extended to achieve higher stability. This incorporated the explicit evaluation of the term *dU* without making it a new variable. This is also known as the second Bassi – Rebay (BR2) method^12^. There are numerous other methods^13^ to tackle these type of equation systems and can also be generalized as the Local Discontinuous Galerkin (LDG) methods^14^. It should also be noted that different methods have been implemented on the DG framework. Some of these methods include Spectral DG method and *hp* – adaptive methods. The first DG spectral method was conducted for elliptic problems^15^ and linear hyperbolic problems^16^. It was further studied for advection diffusion problems, compressible flow and complex geometries^17–19^. Implementation of adaptive methods in DG is straight forward. This is because there is no inter – element continuity requirement, which allows for simple changes of the element order based on the gradient. Lower orders are achieved by making the higher order terms zero. This method has been applied to both hyperbolic conservation laws^20^ and convection diffusion problems^21,22^.

The entire DG framework was implemented in an in-house code called the Multiscale Ionized Gas (MIG) flow code. This is a FORTRAN 90 modular code, which can be used to solve various problems like plasma drift diffusion equations^23^, hypersonic non-equilibrium flow^24^, magnetohydrodynamic equations^25^, and subsonic turbulent flow control^26^. The framework is parallelized via the message passing interface (MPI), which enables it to perform computations on multiple nodes on conventional supercomputing clusters. The sections ahead, will describe the space and time discretization for the Discontinuous Galerkin finite element framework, convergence study, implementation of slope limiters, and parallelization of the code.

### Discontinuous Galerkin space discretization

To understand the discretization process for convection – diffusion problems, a generic scalar equation is chosen which can be extended to any equation system. This is given by

6 $$\frac{\partial U}{\partial t}+\nabla \cdot {\mathop{F}\limits^{\rightharpoonup }}^{inv}(U)-\nabla \cdot {\mathop{F}\limits^{\rightharpoonup }}^{v}(U,\nabla U)=0$$

7 $$U({\boldsymbol{x}},0)={U}_{0}({\boldsymbol{x}})$$

Where *U* denotes the conserved scalar variable, *F*^*inv*^ and *F*^*v*^ denote the inviscid and viscous fluxes respectively and ***x*** ∈ *Ω*, which is the multidimensional domain. All the boundaries are considered periodic in this section. For an element, the approximate solution *U*_*h*_ (***x***, *t*) is represented by Eq. (8).

8 $${U}_{h}(x,t)=\mathop{\sum }\limits_{l=0}^{p}{U}_{K}^{l}(t){\phi }_{l}(x)$$

Where subscript *K* denotes the element, ${U}_{K}^{l}$ denotes the modal degrees of freedom of that element, *φ*_*l*_ denotes the basis function. Legendre polynomials are chosen as the local basis functions because of their property of L^2^ – orthogonality, which leads to a diagonal mass matrix and is beneficial when performing explicit calculations. The list of basis functions for a transformed coordinate system of $x,y,z\in [-1,1]$ is provided in Table 1.

**Table 1:** Basis functions.

Order	*φ*_*l*_(*x*)	*φ*_*l*_(*x*, *y*)	*φ*_*l*_(*x*, *y*, *z*)
0	1	1	1
1	*x*	*x, y*	*x*, *y*, *z*
2	3*x*^2^ − 1	3*x*^2^ − 1, 3*y*^2^ − 1, *xy*	3*x*^2^ − 1, 3*y*^2^ − 1, 3*z*^2^ − 1, *xy*, *yz*, *xz*
3	5*x*^3^ − 3*x*	5*x*^3^ − 3*x*, 5*y*^3^ − 3*y*, (3*x*^2^ − 1)*y*, (3*y*^2^ − 1)*x*	5*x*^3^ − 3*x*, 5*x*^3^ − 3, 5*x*^3^ − 3, (3*x*^2^ − 1)*y*, (3*y*^2^ − 1)*z*, (3*y*^2^ − 1)*x*, (3*y*^2^ − 1)*z*, (3*z*^2^ − 1)*x*, (3*z*^2^ − 1)*y*

To obtain the weak form of the equation, the variable *U* is replaced by *U*_*h*_ and Eq. (6) is multiplied with the basis function *φ*_*l*_. After integration by parts, Eq. (9) is obtained.

9 $$\begin{array}{c}\frac{d}{dt}{\int }_{K}{U}_{h}\phi (x)\,dx-{\int }_{K}{\mathop{F}\limits^{\rightharpoonup }}^{inv}\cdot \nabla \phi (x)dx+\sum _{e\in \Gamma }{\int }_{e}{\mathop{F}\limits^{\rightharpoonup }}^{inv}\cdot {\mathop{n}\limits^{\rightharpoonup }}_{e,K}\phi (x)d\Gamma \\ +{\int }_{K}{\mathop{F}\limits^{\rightharpoonup }}^{v}\cdot \nabla \phi (x)dx-\sum _{e\in \Gamma }{\int }_{e}{\mathop{F}\limits^{\rightharpoonup }}^{v}\cdot {\mathop{n}\limits^{\rightharpoonup }}_{e,K}\phi (x)d\Gamma =0\end{array}$$

**Figure 1:**
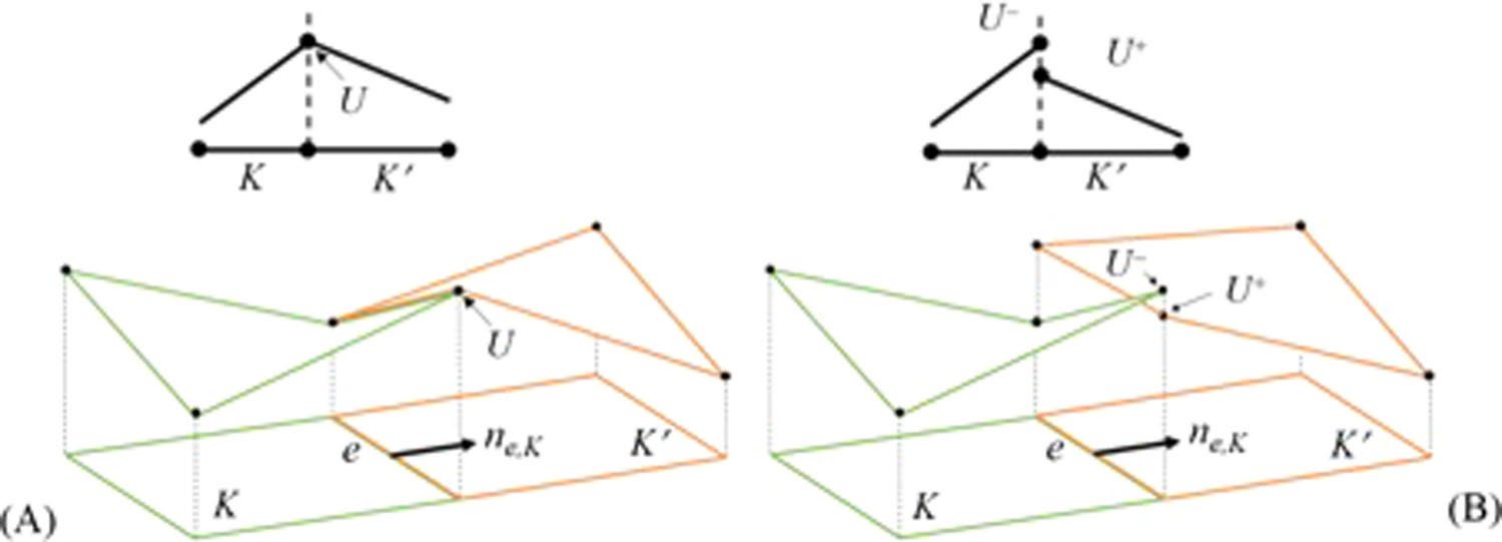
Comparison between continuous and discontinuous Galerkin method. (**A**) Continuous element with interface solution *U* for element *K* and *K*′ and (**B**) discontinuous element with interface solutions *U*^−^ and *U*^+^ for element *K* and *K*′ respectively sharing the edge *e* with an outward unit normal ${n}_{e,K}$.

In Eq. (9), *n*_*e*,*K*_ denotes the outward unit normal for the edge *e* (it can be a face or an edge) of element *K*. Figure 1 shows a representation of these elements. The element boundary space is denoted by Γ. For the terms in summation, where fluxes are to be evaluated at the element interfaces, the solution *U*_*h*_ is discontinuous and cannot be uniquely defined. Thus, the terms must be replaced by a locally Lipschitz, consistent, monotone flux to maintain the stability and convergence properties of the scheme with higher order of accuracy^8^. In Eq. (9), ${\mathop{F}\limits^{\rightharpoonup }}^{v}$ is a function of both *U* and ∇*U*, which implies that either ∇*U* needs to be evaluated as a new variable or treated explicitly. Detailed descriptions of the numerical integration, fluxes and terms are provided in the next two sections.

### Numerical integration

All the integrals can be written in discrete form using Gauss – Legendre quadrature rules.

10 $${\int }_{K}f(x)dx=jac{\int }_{-1}^{1}f(x^{\prime} )dx^{\prime} =jac\mathop{\sum }\limits_{n=1}^{N}{w}_{n}\,f({x^{\prime} }_{n})dx^{\prime} $$

In Eq. (10) *jac* is obtained when transforming from global coordinate system to local coordinate system. Also for all the integrals shown, the basis functions vary with space, while the degrees of freedom vary in time as shown in Eq. (8). Since the basis functions are already in transformed space ${x^{\prime} }_{n}$ are the Gauss – Legendre points provided in Table 2. One should note that for multidimensional integration the single summation becomes multiple summations with quadrature points ${x^{\prime} }_{n}$ and weights *w*_*n*_ being obtained via tensor product of one-dimensional weights and points.

**Table 2:** Gauss – Legendre Quadrature.

*n*	1	2	3		4		5		
*w*_*n*_	2	1	$\,\frac{8}{9}$	$\frac{5}{9}$	$\,\frac{18+\sqrt{30}}{36}$	$\frac{18-\sqrt{30}}{36}$	$\frac{128}{225}$	$\frac{322+13\sqrt{70}}{900}$	$\frac{322-13\sqrt{70}}{900}$
${x^{\prime} }_{n}$	0	$\pm \sqrt{\frac{1}{3}}$	0	$\pm \sqrt{\frac{3}{5}}$	$\pm \sqrt{\frac{3}{7}-\frac{2}{7}\sqrt{\frac{6}{5}}}$	$\,\pm \sqrt{\frac{3}{7}+\frac{2}{7}\sqrt{\frac{6}{5}}}$	0	$\,\pm \sqrt{\frac{5}{9}-\frac{2}{9}\sqrt{\frac{10}{7}}}$	$\,\pm \sqrt{\frac{5}{9}+\frac{2}{9}\sqrt{\frac{10}{7}}}$

### Inviscid fluxes

As mentioned earlier, the discontinuity at the element interfaces requires the use of numerical fluxes. There are wide variety of numerical fluxes which satisfy the locally Lipschitz, monotone and consistent criteria^27^. However, the present work uses either Godunov flux or Local Lax-Friedrichs flux^28^. The later, also known as ENO-LLF, provides better shock capturing with improved accuracy. Although it is more diffusive than the Roe flux and the Godunov flux, its impact on the solution is insignificant for higher order approximations^27^. After replacing the inviscid flux in Eq. (9) with the numerical flux ${h}_{e,K}^{inv}$, the first summation term can be written as

11 $$\sum _{e\in \Gamma }{\int }_{e}{\mathop{F}\limits^{\rightharpoonup }}^{inv}\cdot {\mathop{n}\limits^{\rightharpoonup }}_{e,K}\phi (x)d\Gamma =\sum _{e\in \Gamma }{\int }_{e}{h}_{e,K}^{inv}({U}_{h}^{-},{U}_{h}^{+})\phi (x)d\Gamma $$

The + and − states of the solution refer to the outside and inside solution along edge *e* as depicted in Fig. 1. The Godunov flux is given by

12 $${h}_{e,K}^{inv}({U}^{-},{U}^{+})=\{\begin{array}{c}{{\rm{\min }}}_{{U}^{-}\le U\le {U}^{+}}{F}^{inv}(U),\,{\rm{if}}\,{U}^{-}\le {U}^{+}\\ {{\rm{\max }}}_{{U}^{+}\le U\le {U}^{-}}{F}^{inv}(U),\,{\rm{otherwise}}\end{array}\}$$

Eq. (12) can be interpreted as, if the neighbouring solution *U*^+^ is larger than the inside solution *U*^−^ then choose the minimum flux $({\rm{\min }}\,[{F}^{inv}({U}^{+}),\,{F}^{inv}({U}^{-})])$ otherwise choose the maximum of the two.

The Lax – Friedrichs flux is given by

13 $${h}_{e,K}^{inv}({U}_{h}^{+},{U}_{h}^{-})=\frac{1}{2}[{\mathop{F}\limits^{\rightharpoonup }}^{inv}({U}_{h}^{-})\cdot {\mathop{n}\limits^{\rightharpoonup }}_{e,K}+{\mathop{F}\limits^{\rightharpoonup }}^{inv}({U}_{h}^{+})\cdot {\mathop{n}\limits^{\rightharpoonup }}_{e,K}-{\alpha }_{e,K}({U}_{h}^{+}-{U}_{h}^{-})]$$

In Eq. (13) *α*_*e*,*K*_ is obtained by evaluating the largest absolute eigenvalue of the jacobian matrices for the outside and inside elements.

14 $$\begin{array}{c}{\alpha }_{e,K}=\,{\rm{\max }}[\{abs({{\boldsymbol{\lambda }}}^{+})\},\{abs({{\boldsymbol{\lambda }}}^{-})\}]\\ {{\boldsymbol{\lambda }}}^{+}=eigenvalue\{\frac{\partial F({\bar{U}}^{+})}{\partial U}\cdot {n}_{e,K}\}\\ {{\boldsymbol{\lambda }}}^{-}=eigenvalue\{\frac{\partial F({\bar{U}}^{-})}{\partial U}\cdot {n}_{e,K}\}\end{array}$$

For Euler equations or Navier-Stokes equations the eigenvalues are *u* + *a*, *u* − *a* and *u*, where *a* is the speed of sound. In Eq. (14), $\bar{U}$ is the mean solution of the inside or outside element depending on the *λ* being evaluated.

### Viscous fluxes

The viscous terms in Eq. (9) can be modelled in numerous ways. Some of the common methods are LDG^14^, Bassi – Rebay (BR1 and BR2)^11,12^, Interior Penalty (IP)^29^, Baumann – Oden^21^ etc. type methods. A detailed comparison and insight on these methods can be found in Arnold *et al*.^13^. However, for brevity only the LDG, BR1 and BR2 schemes are described here.

The viscous fluxes include ∇*U* as an unknown which must be evaluated either a priori or along with the equation system. To evaluate ∇*U*, Eq. (6) is first changed to Eq. (15) and Eq. (16).

15 $$\frac{\partial U}{\partial t}+\nabla \cdot {\mathop{F}\limits^{\rightharpoonup }}^{inv}(U)-\nabla \cdot {\mathop{F}\limits^{\rightharpoonup }}^{v}(U,\theta )=0$$

16 $$\theta =\nabla U$$

The same procedure as mentioned before is followed and finally equations like Eq. (9) are obtained.

17 $$\begin{array}{c}\frac{d}{dt}{\int }_{K}{U}_{h}\phi (x)dx-{\int }_{K}{\mathop{F}\limits^{\rightharpoonup }}^{inv}({U}_{h})\cdot \nabla \phi (x)dx+\sum _{e\in \Gamma }{\int }_{e}{\mathop{F}\limits^{\rightharpoonup }}^{inv}({U}_{h})\cdot {\mathop{n}\limits^{\rightharpoonup }}_{e,K}\phi (x)d\Gamma \\ \,+{\int }_{K}{\mathop{F}\limits^{\rightharpoonup }}^{v}({U}_{h},{\theta }_{h})\cdot \nabla \phi (x)dx-\sum _{e\in \Gamma }{\int }_{e}{\mathop{F}\limits^{\rightharpoonup }}^{v}({U}_{h},{\theta }_{h})\cdot {\mathop{n}\limits^{\rightharpoonup }}_{e,K}\phi (x)d\Gamma =0\end{array}$$

18 $${\int }_{K}{\theta }_{h}\phi (x)dx+{\int }_{K}{U}_{h}\nabla \phi (x)dx-\sum _{e\in \Gamma }{\int }_{e}{U}_{h}{\mathop{n}\limits^{\rightharpoonup }}_{e,K}\phi (x)d\Gamma =0$$

It should be noted that in Eq. (17) and Eq. (18) *θ*_*h*_ denotes the approximate solution of the auxiliary variable *θ* as in the definition given in Eq. (8). As discussed earlier, the discontinuous interface requires the fluxes in the summation terms to be evaluated using a locally Lipschitz, consistent and monotone flux. Therefore the last terms in Eq. (17) and Eq. (18) are represented as Eq. (19) and Eq. (20).

19 $$\sum _{e\in \Gamma }\,{\int }_{e}{\mathop{F}\limits^{\rightharpoonup }}^{v}({U}_{h},{\theta }_{h})\cdot {\mathop{n}\limits^{\rightharpoonup }}_{e,K}\phi (x)d\Gamma =\sum _{e\in \Gamma }{\int }_{e}{h}_{e,K}^{v}({U}_{h}^{+},{U}_{h}^{-},{\theta }_{h}^{+},{\theta }_{h}^{-})\cdot {\mathop{n}\limits^{\rightharpoonup }}_{e,K}\phi (x)d\Gamma $$

20 $$\sum _{e\in \Gamma }{\int }_{e}{U}_{h}{\mathop{n}\limits^{\rightharpoonup }}_{e,K}\phi (x)\,d\Gamma =\sum _{e\in \Gamma }{\int }_{e}{h}_{e,K}^{\theta }({U}_{h}^{+},{U}_{h}^{-},{\theta }_{h}^{+},{\theta }_{h}^{-}){\mathop{n}\limits^{\rightharpoonup }}_{e,K}\phi (x)d\Gamma $$

The choice of numerical fluxes ${h}_{e,K}^{v}$ and ${h}_{e,K}^{\theta }$ gives rise to different methods.

### Local discontinuous Galerkin method

The viscous numerical fluxes for this method can be written as

21 $$\begin{array}{c}h({U}_{h}^{+},{U}_{h}^{-},{\theta }_{h}^{+},{\theta }_{h}^{-})=\frac{1}{2}[{\mathop{F}\limits^{\rightharpoonup }}^{v}({U}_{h}^{+},{\theta }_{h}^{+})+{\mathop{F}\limits^{\rightharpoonup }}^{v}({U}_{h}^{-},{\theta }_{h}^{-})+{\boldsymbol{C}}({{\boldsymbol{U}}}^{+}-{{\boldsymbol{U}}}^{-})]\\ {\boldsymbol{C}}=[\begin{array}{cc}{c}_{11} & {c}_{12}\\ -{c}_{12} & 0\end{array}],{\boldsymbol{U}}=\{U,\theta \}\end{array}$$

Using Eq. (21) and since ${\mathop{F}\limits^{\rightharpoonup }}^{v}$ for Eq. (16) is *U*, obtain the expressions for ${h}_{e,K}^{v}$ and ${h}_{e,K}^{\theta }$ are

22 $$\begin{array}{c}{h}_{e,K}^{v}({U}_{h}^{+},{U}_{h}^{-},{\theta }_{h}^{+},{\theta }_{h}^{-})=\frac{1}{2}[{\mathop{F}\limits^{\rightharpoonup }}^{v}({U}_{h}^{+},{\theta }_{h}^{+})+{\mathop{F}\limits^{\rightharpoonup }}^{v}({U}_{h}^{-},{\theta }_{h}^{-})]+{c}_{11}({U}_{h}^{+}-{U}_{h}^{-})+{c}_{12}({\theta }_{h}^{+}-{\theta }_{h}^{-})\\ {h}_{e,K}^{\theta }({U}_{h}^{+},{U}_{h}^{-},{\theta }_{h}^{+},{\theta }_{h}^{-})=\frac{1}{2}({U}_{h}^{+}+{U}_{h}^{-})-{c}_{12}({U}_{h}^{+}-{U}_{h}^{-})\end{array}$$

A detailed discussion about the choice of constants *c*_11_ and *c*_12_, as well as the extension to multidimensional problems have been described by Cockburn and Shu^14^.

### Bassi – Rebay method I

The numerical fluxes ${h}_{e,K}^{v}$ and ${h}_{e,K}^{\theta }$ are obtained by averaging the fluxes at the edge of the element and its neighbor. This is provided in Eq. (23) and Eq. (24)

23 $${h}_{e,K}^{v}({U}_{h}^{+},{U}_{h}^{-},{\theta }_{h}^{+},{\theta }_{h}^{-})=\frac{1}{2}[{\mathop{F}\limits^{\rightharpoonup }}^{v}({U}_{h}^{+},{\theta }_{h}^{+})+{\mathop{F}\limits^{\rightharpoonup }}^{v}({U}_{h}^{-},{\theta }_{h}^{-})]$$

24 $${h}_{e,K}^{\theta }({U}_{h}^{+},{U}_{h}^{-},{\theta }_{h}^{+},{\theta }_{h}^{-})=\frac{1}{2}[{U}_{h}^{+}+{U}_{h}^{-}]$$

The above method describes the BR1 scheme. However due to the method’s deficiencies, such as non – optimal accuracy for purely elliptic problems, spread stencil and increase in the number of degrees of freedom per element (specially for implicit algorithm)^12^, lead to the implementation of BR2 scheme.

### Bassi – Rebay method II

This scheme uses the property that, the evaluation of a solution gradient inside the element is trivial and can be obtained using the gradients of the basis functions. However, for *P* = 0 elements and at interface discontinuities it is not trivial. To obtain ∇*U* without adding an extra equation a correction term *R* is added. This is known as the lift operator. After few mathematical manipulations^12^ Eq. (18) can be rewritten as Eq. (25).

25 $${\int }_{K}{\theta }_{h}\phi (x)dx={\int }_{K}\phi (x)\nabla {U}_{h}dx+\sum _{e\in \Gamma }{\int }_{e}\frac{1}{2}({U}_{h}^{+}+{U}_{h}^{-}){\mathop{n}\limits^{\rightharpoonup }}_{e,K}\phi (x)d\Gamma $$

Thus, we can write *θ*_*h*_ = ∇*U*_*h*_ + *R*_*h*_, where *R*_*h*_ is defined like Eq. (8) and can be obtained using Eq. (26).

26 $${\int }_{K}{R}_{h}\phi (x)dx=\sum _{e\in \Gamma }{\int }_{e}\frac{1}{2}({U}_{h}^{+}+{U}_{h}^{-}){\mathop{n}\limits^{\rightharpoonup }}_{e,K}\phi (x)d\Gamma $$

Using the global lifting operator leads to a non-compact stencil which can be avoided by using local lift operators *r*_*h*_. This is defined by

27 $${\int }_{K}{r}_{h}\phi \,(x)dx={\int }_{e}\frac{1}{2}({U}_{h}^{+}+{U}_{h}^{-}){\mathop{n}\limits^{\rightharpoonup }}_{e,K}\phi (x)d\Gamma ,\,{R}_{h}=\sum _{e\in K}{r}_{h}$$

When performing volume integrals, global lift operators are used and for element boundary integrals, local lift operators are used. Using this scheme leads to a reduction in the number of degrees of freedom. The information from immediate neighbors is only required producing a compact stencil. This minimization of information needed from the local region means that the method spends most of its time computing local integrals, and the communication workload is far smaller than the computational workload. A scenario then arises where most of the calculations in each individual element are independent and thus almost “embarrassingly parallel” making them amenable to exploit maximum parallel efficiencies.

### Temporal discretization

The choice of time integration depends on the problem in hand. For transient accuracy, high order time accurate schemes need to be implemented. Problems involving acoustic wave propagation fall in this category. This section will describe some of the common time integration methods implemented and their advantages and disadvantages.

#### Explicit time integration

To solve the nonlinear hyperbolic conservation laws in a DG framework an explicit implementation of the method was introduced^30^. This overcame the issue of solving nonlinear problems on an element by element basis. However, an explicit method is restricted by the CFL condition. To improve the stability of the scheme a TVDM slope limiter was implemented^31^. However, this method was only first order accurate in time and the slope limiter affected the smooth regions of the solution reducing the spatial accuracy. This was finally overcome by using the RKDG method and a modified slope limiter which was second order in time and maintained the accuracy of the scheme in smooth regions^7^. This made the scheme stable for CFL ≤ 1/3. To show the explicit time integration Eq. (9) is written in a modified form given by Eq. (29).

$$\frac{d}{dt}{\int }_{K}{U}_{h}(x,{t}^{n})\phi (x)dx={L}_{h}[{U}_{h}(x,{t}^{n})]$$

$$\frac{d}{dt}{\int }_{K}{U}_{K}^{l}({t}^{n}){\phi }_{l}(x){\phi }_{l}^{T}(x)dx={L}_{h}[{U}_{h}(x,{t}^{n})]$$

28 $$\frac{d}{dt}[{U}_{K}^{l}({t}^{n})]{\int }_{K}{\phi }_{l}(x){\phi }_{l}^{T}(x)dx=\frac{d}{dt}[{U}_{K}^{l}({t}^{n})][M]={L}_{h}[{U}_{h}(x,{t}^{n})]$$

29 $$\frac{d}{dt}[{U}_{K}^{l}({t}^{n})]={L}_{h}[{U}_{h}(x,{t}^{n})]{[M]}^{-1}$$

The mass matrix [*M*], is diagonal for the present choice of basis functions. For the simple Euler explicit case, Eq. (29) can be written as Eq. (30) which yields first order accuracy in time.

30 $$[{U}_{K}^{l}({t}^{n+1})-{U}_{K}^{l}({t}^{n})]=(\Delta t){L}_{h}[{U}_{h}(x,{t}^{n})]{[M]}^{-1}$$

Using the second order RKDG method the solution can be more time accurate. This is described in Eq. (31)

$$[{U}_{K}^{l}({t}^{m})]=[{U}_{K}^{l}({t}^{n})]\,+(\Delta t){L}_{h}[{U}_{h}(x,{t}^{n})]{[M]}^{-1}$$

$$[{U}_{K}^{l}({t}^{m+1})]=[{U}_{K}^{l}({t}^{m})]\,+(\Delta t){L}_{h}[{U}_{h}(x,{t}^{m})]{[M]}^{-1}$$

31 $$[{U}_{K}^{l}({t}^{n+1})]=\frac{1}{2}([{U}_{K}^{l}({t}^{m+1})+{U}_{K}^{l}({t}^{n})])$$

The RKDG method has been proven to give CFL ≤ 1/3 for *P* = 1 and CFL ≤ 1/5 for *P* = 2 case^7^. Although RKDG scheme has high parallelizability, being an explicit scheme it has CFL restrictions.

#### Implicit time integration

Since the problems studied are nonlinear in nature, the Newton’s method is employed to solve for the equation system. The goal here is to find a value iteratively, which would be closest to the actual solution. Thus, Eq. (29) is written as Eq. (32) for iteration *q*

32 $$f({U}_{K}^{l}({t}^{n,q}))=\frac{d}{dt}[{U}_{K}^{l}({t}^{n,q})]-{L}_{h}[{U}_{h}(x,{t}^{n,q})]{[M]}^{-1}\approx 0$$

To get the next time step solution Eq. (32) is discretized in time using the Euler Implicit algorithm to obtain Eq. (33).

33 $$f({U}_{K}^{l}({t}^{n+1,q}),{U}_{K}^{l}({t}^{n+1,q-1}))=[{U}_{K}^{l}({t}^{n+1,q})-{U}_{K}^{l}({t}^{n+1,q-1})]-\Delta t{L}_{h}[{U}_{h}(x,{t}^{n+1,q})]{[M]}^{-1}$$

Therefore, for *q* ≥ 1, Newton’s method can be applied to Eq. (33). It should be noted that when *q* = 1 in Eq. (33), ${U}_{K}^{l}({t}^{n+1,q-1})={U}_{K}^{l}({t}^{n})$.

34 $$[\frac{\partial \{f({U}_{K}^{l}({t}^{n+1,q}),{U}_{K}^{l}({t}^{n,q}))\}}{\partial {U}_{K}^{l}({t}^{n+1,q})}][{U}_{K}^{l}({t}^{n+1,q+1})-{U}_{K}^{l}({t}^{n+1,q})]=-\,f({U}_{K}^{l}({t}^{n+1,q+1}),{U}_{K}^{l}({t}^{n+1,q}))$$

## Test Cases

### Taylor green vortex

#### Background

This is one of the canonical problems studied for hydrodynamic turbulence. This has been extensively studied in literature to derive empirical and analytical relations in turbulent flow physics. Early in depth numerical investigation of this problem was done by Orszag^32^ and Brachet *et al*.^33,34^. This problem was also studied by Comte-Bellot and Corrsin^35^ experimentally as a grid turbulence problem. These studies have become the benchmark for turbulent code validation. Since then, different numerical methods^36–39^ have been used to improve or validate these studies. Results for different Reynolds number, mesh and spatial order of accuracy are compared and investigated. The domain size $\,\Omega =(2\pi \times 2\pi \times 2\pi )$ with periodic boundaries on all faces. The initial conditions for this problem are

$${u}_{0}=\,\sin (x)\,\cos (y)\,\cos (z),\,{v}_{0}=\,\sin (y)\,\cos (x)\,\cos (z),\,{w}_{0}=0,$$

35 $${p}_{0}=100+\frac{1}{16}(\cos (2x)+\,\cos (2y))(\cos (2z)+2),{\rho }_{0}=1\,$$

This problem is solved using RKDG method, which involves RK2 time marching and LDG scheme for viscous flux. Two types of inviscid fluxes are tested, namely Godunov flux and LLF flux. The mesh is uniform in all directions and the DOFs for an *N*^3^ mesh corresponds to ${N}^{3}\times {(P+1)}^{3}$. Although the cases can be run at different time step Δ*t*, the solutions are obtained using Δ*t* = 2.5 × 10^−4^, to have similar time diffusion. The time step is kept low since the Godunov flux requires more restrictive time stepping than the LLF flux. The simulations are run till *t* = 10. Three main parameters are used to study this case. These include the integrated kinetic energy *E*_*k*_, kinetic energy dissipation rate *ε* and integrated enstrophy *ζ*. These parameters are given in Eq. (36). For incompressible flows *ε* and *ζ* can be related using the relation given in Eq. (37). It should be noted that evaluation of *ε* (*ζ*) requires additional degrees of freedom to reach the correct *ε* levels when compared to *ε* (*E*_*k*_).

36 $${E}_{k}=\frac{1}{{\rho }_{0}\Omega }{\int }_{\Omega }\rho \frac{{\bf{v}}\cdot {\bf{v}}}{2}d\Omega \,;\,\varepsilon ({E}_{k})=-\,\frac{\partial {E}_{k}}{\partial t};\,\zeta =\frac{1}{{\rho }_{0}\Omega }{\int }_{\Omega }\rho \frac{{\boldsymbol{\omega }}\cdot {\boldsymbol{\omega }}}{2}d\Omega \,$$

37 $$\varepsilon (\zeta )=\frac{2\zeta }{{\rm{Re}}}$$

#### Effect of Reynolds number

**Figure 2:**
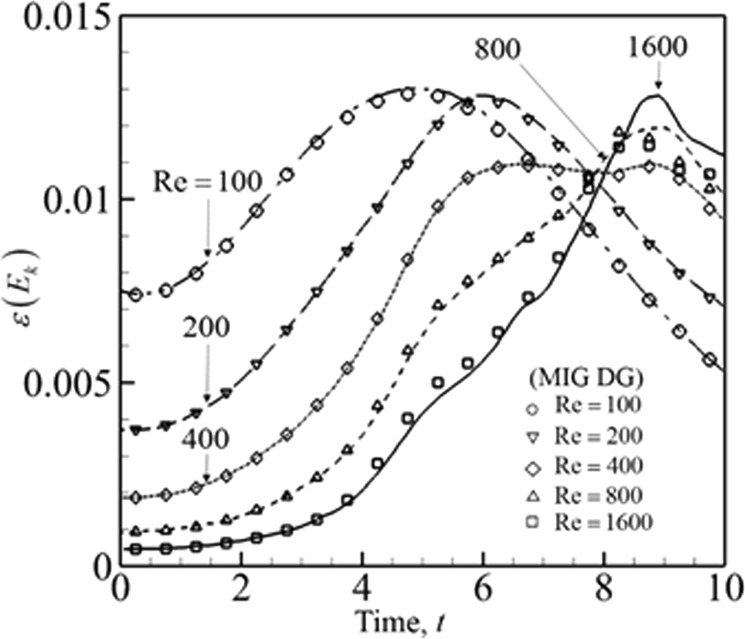
Energy dissipation rate at different Reynolds number using third order accurate DG solution on a 60^3^ mesh compared with DNS results^34^.

To study the effect of Reynolds number (Re), the inviscid flux is kept as Godunov flux and a 60^3^ (180^3^ degrees of freedom) mesh size is used. The third order accurate (*P* = 2) spatial accuracy is chosen. The Reynolds numbers tested are 100, 200, 400, 800 and 1600. The normalized Root Mean Square (RMS) error of $\varepsilon ({E}_{k})$ in comparison with DNS data is given in Table 3. The norm error is evaluated using Eq. (38). The timestep is 10^−3^ sec and data is printed at every 250 steps within the 10 sec interval (*N* = 40) for all cases considered for Eq. (38). Except Re = 1600 all the other Reynolds number have results that are comparative to DNS results^34^. The profile of kinetic energy dissipation rate *ε* (*E*_*k*_) is shown in Fig. 2. The dissipation rate is captured accurately by MIG DG ILES. However, in the next section it will be seen that using LLF inviscid flux has slightly more error than the Godunov flux due to its higher dissipation.

38 $${\rm{Norm}}\,{\rm{RMS}}\,{\rm{Error}}=\sqrt{\frac{{\sum }_{i=1}^{N}{({\varepsilon }_{i}-{\varepsilon }_{{\rm{DNS}}})}^{2}}{N}}$$

**Table 3:** Norm RMS Error in dissipation rate at different Reynolds number.

Re	Norm RMS Error
100	2.25 × 10^−6^
200	2.85 × 10^−6^
400	2.62 × 10^−6^
800	3.14 × 10^−5^
1600	3.43 × 10^−4^

#### Effect of inviscid numerical flux

**Figure 3:**
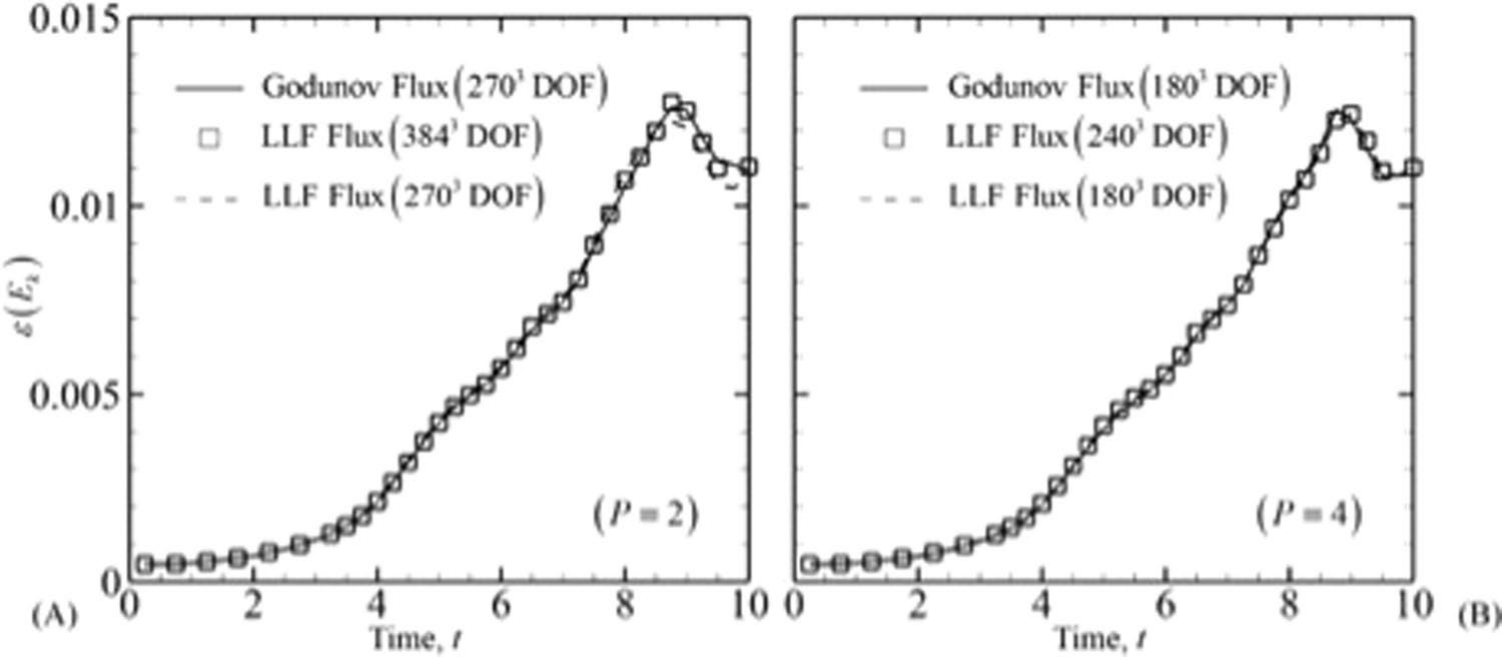
Comparison of energy dissipation rate for different inviscid numerical fluxes at different degrees of freedom and polynomial order. Dissipation rate for (**A**) *P* = 2 and (**B**) *P* = 4.

To study the effect of numerical fluxes, the Godunov flux and LLF flux are tested for a 60^3^ with *P* = 2 (180^3^ DOF), 45^3^ with *P* = 3 (180^3^ DOF) and 36^3^ with *P* = 4 (180^3^ DOF) mesh sizes. It should be noted that the total DOF is calculated by *N*^3^ × (*P* + 1)^3^. The Reynolds number for the cases here is kept at Re = 1600. The normalized RMS error of *ε* (*E*_*k*_) in comparison with DNS data^36^ is given in Table 4. The dissipation rate has higher errors when the LLF scheme is used. The greater diffusive nature of LLF flux was also observed by Beck *et al*.^40^ when comparing with the Roe scheme. However, the differences are very low as the errors are two orders of magnitude lower than the variable value. It should be noted that although Godunov flux is more accurate due to its least dissipative nature, it creates larger oscillations which can result in backscatter and also requires a lower time step. Therefore, although LLF has higher numerical dispersion, it is preferable to be used with slightly higher degrees of freedom. For this problem using around 1.4 times the number of DOF in each direction matches the solutions for both the fluxes at *P* = 2. For higher orders, the differences in dissipation rate due to fluxes become negligible. This can be observed in Fig. 3 which depicts the similarity in solutions for the two fluxes at different degrees of freedom for a *P* = 2 and *P* = 4 case.

**Table 4:** Norm RMS Error in dissipation rate for Godunov and LLF fluxes.

Order	Godunov Flux	Local Lax – Friedrichs flux
2	3.43 × 10^−4^	7.35 × 10^−4^
3	9.38 × 10^−5^	3.36 × 10^−4^
4	7.83 × 10^−5^	1.93 × 10^−5^

#### Effect of spatial order of accuracy

**Figure 4:**
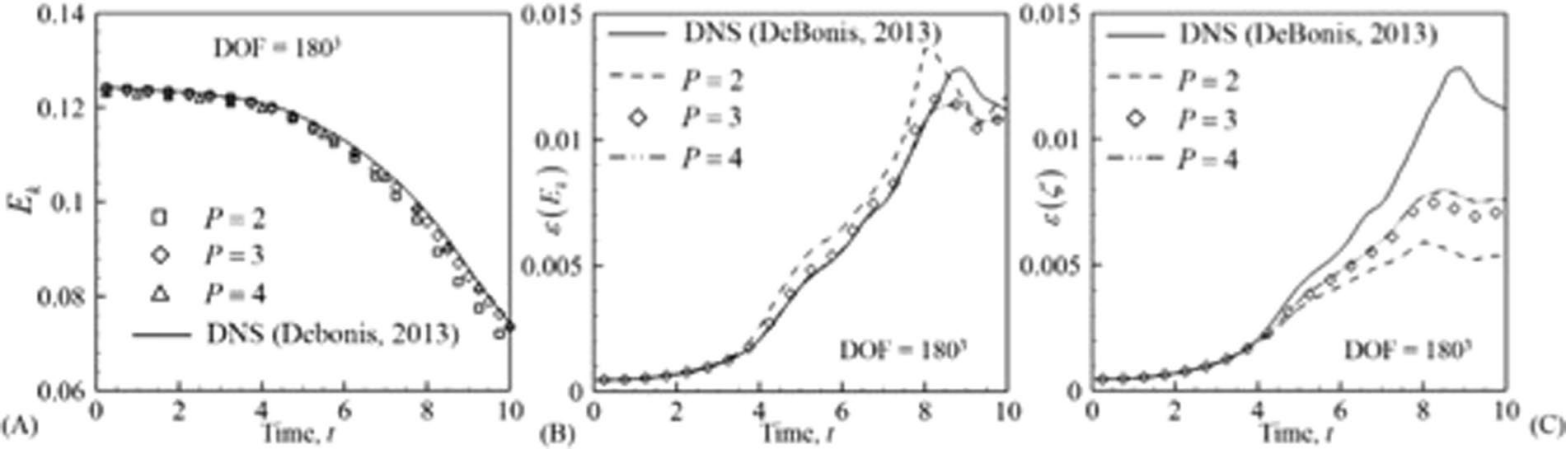
Comparison of the MIG DG solution with published DNS results^36^. (**A**) Turbulent kinetic energy, (**B**) energy dissipation rate based on integral kinetic energy and (**C**) energy dissipation rate based on enstrophy.

To study the effect of spatial order of accuracy LLF flux is chosen as the inviscid numerical flux. The problem is studied using orders *P* = 2, *P* = 3 and *P* = 4. The Reynolds number for the cases here is kept at Re = 1600. All the parameters mentioned in Eq. (36) and Eq. (37) are depicted in Fig. 4. Both *ε* (*ζ*) and *ε* (*E*_*k*_) are compared to highlight the differences between ILES results and DNS results^36^ as well as to show that, capturing gradients in ILES requires more degrees of freedom. The DNS results are obtained using 13-point DRP scheme with 512^3^ grid. The solutions obtained using *P* = 2 have the largest error for the same DOF. This is a known property which is utilized in turbulent flow simulations using higher order methods. However, as shown in the previous paragraph, the differences between the fluxes are negligible.

**Figure 5:**
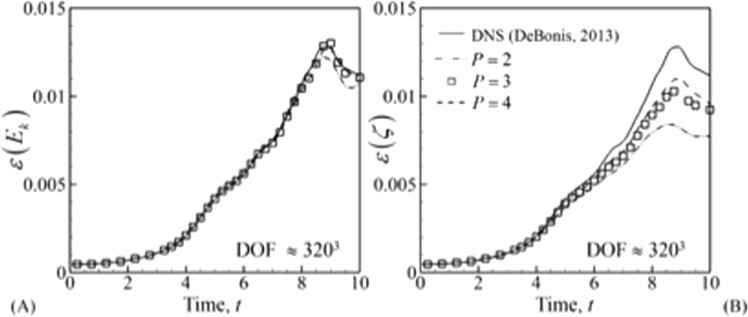
Comparison of turbulent kinetic energy dissipation rate for different order of spatial accuracy at approximately 320^3^ DOF. (**A**) Dissipation rate based on integral kinetic energy and (**B**) enstrophy.

To see if the solution converges, higher DOFs were compared to the DNS solution. This is depicted in Fig. 5. Although *ε* (*E*_*k*_) has converged to the DNS solution, *ε* (*ζ*) has not converged yet. This behavior was also observed by DeBonis^36^ who performed a comparison between 4^th^, 8^th^ and 12^th^ order central finite difference schemes with a 13-point DRP scheme (DNS). Similar behavior has been found for DNS^41^ solutions using DG methods.

#### Energy spectrum

**Figure 6:**
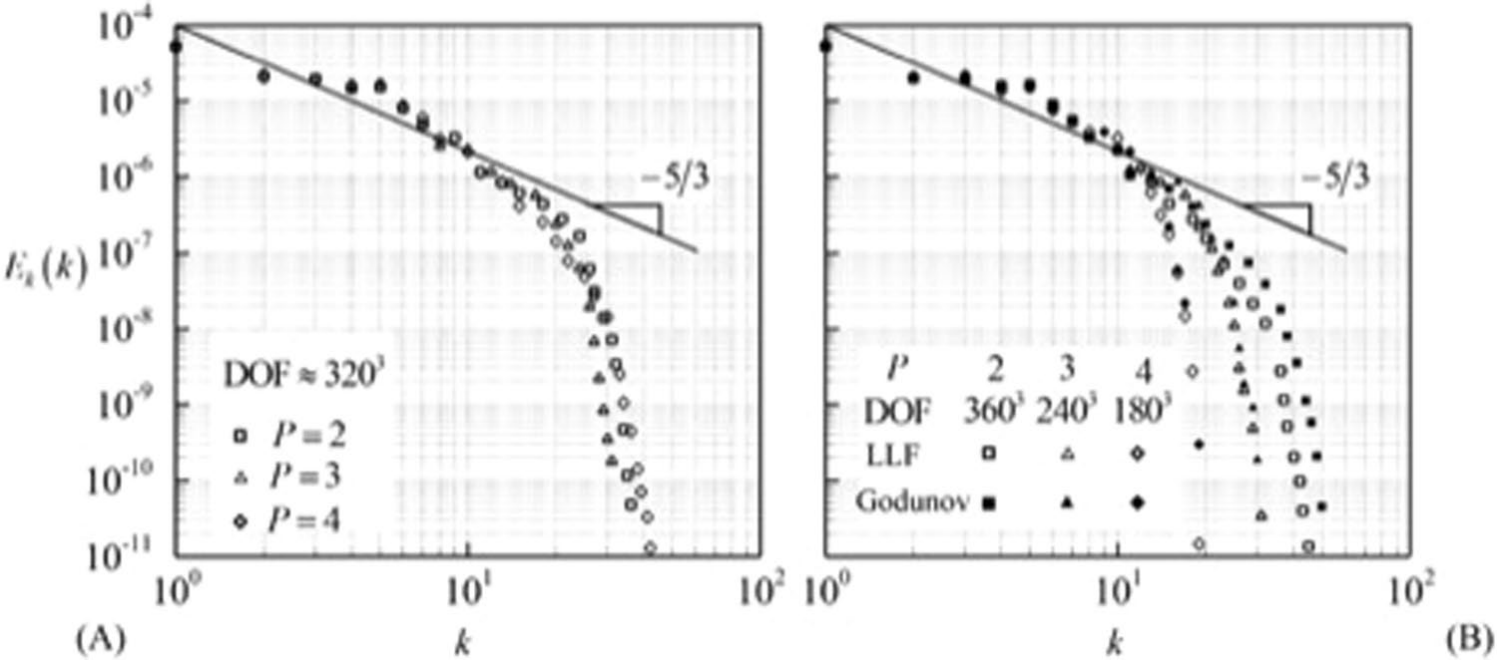
Kinetic energy spectrum for Taylor Green vortex problem at *t* = 10. (**A**) Effect of polynomial order and (**B**) effect of inviscid flux on energy spectrum.

The kinetic energy spectrum for all the cases is plotted at *t* = 10 in Fig. 6. All the curves follow the standard turbulent spectrum of −5/3 slope. The differences between the spectrums for different order polynomials depicted in Fig. 6(A) are negligible. Note, the effect of flux is not significant on the energy spectrum.

#### Flow structures

**Figure 7:**
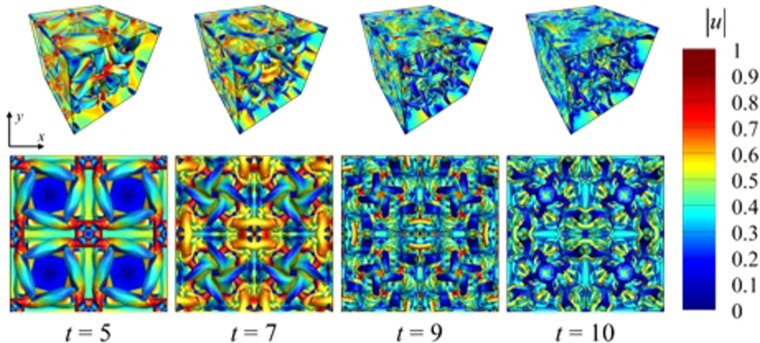
Instantaneous *Q* – criterion colored with velocity magnitude showing breakdown of coherent structures with time for a Taylor Green vortex problem.

The instantaneous iso-surface of *Q* – criterion (positive second scalar invariant of ∇*u*) colored with velocity magnitude is depicted in Fig. 7. The equation defining *Q* – criterion is provided in Eq. (39). The data corresponds to the simulation with *P* = 3 (DOF = 320^3^). The coherent structures keep breaking down into smaller structures as the time progresses and finally around *t* = 9 the flow becomes fully turbulent.

39 $$Q=\frac{1}{2}[{|\Omega |}^{2}-{|{\bf{S}}|}^{2}];\,\Omega =\frac{1}{2}[\nabla {\bf{v}}-{(\nabla {\bf{v}})}^{T}];\,{\bf{S}}=\frac{1}{2}[\nabla {\bf{v}}+{(\nabla {\bf{v}})}^{T}]$$

## Parallel Algorithms

To parallelize the MIG code, open MPI was used and the code was tested at the University of Florida high performance computing center. All the tests were run on servers with Intel E5-2698 v3 processors with the capability to achieve HPL R_*max*_ of 7.381 × 10^5^ GFlops. The domain was decomposed lexicographically with equal elements in each processor. The solution time for N-S equations was studied for processor counts of 1, 8, 16, 32, 64, 128, 256 and 512. The parallel performance is studied by solving the time explicit N-S equations for the Taylor Green vortex isotropic turbulence problem. Two cases were tested with total number of elements, 32^3^ (DOF = 5570560) and 64^3^ (DOF = 44545480). A small number of elements was chosen to result in a partitioning scheme with significant communication time with respect to the calculations performed. The problem is run for 100 time steps to average out the total time duration and the all the tests are repeated three times.

**Figure 8:**
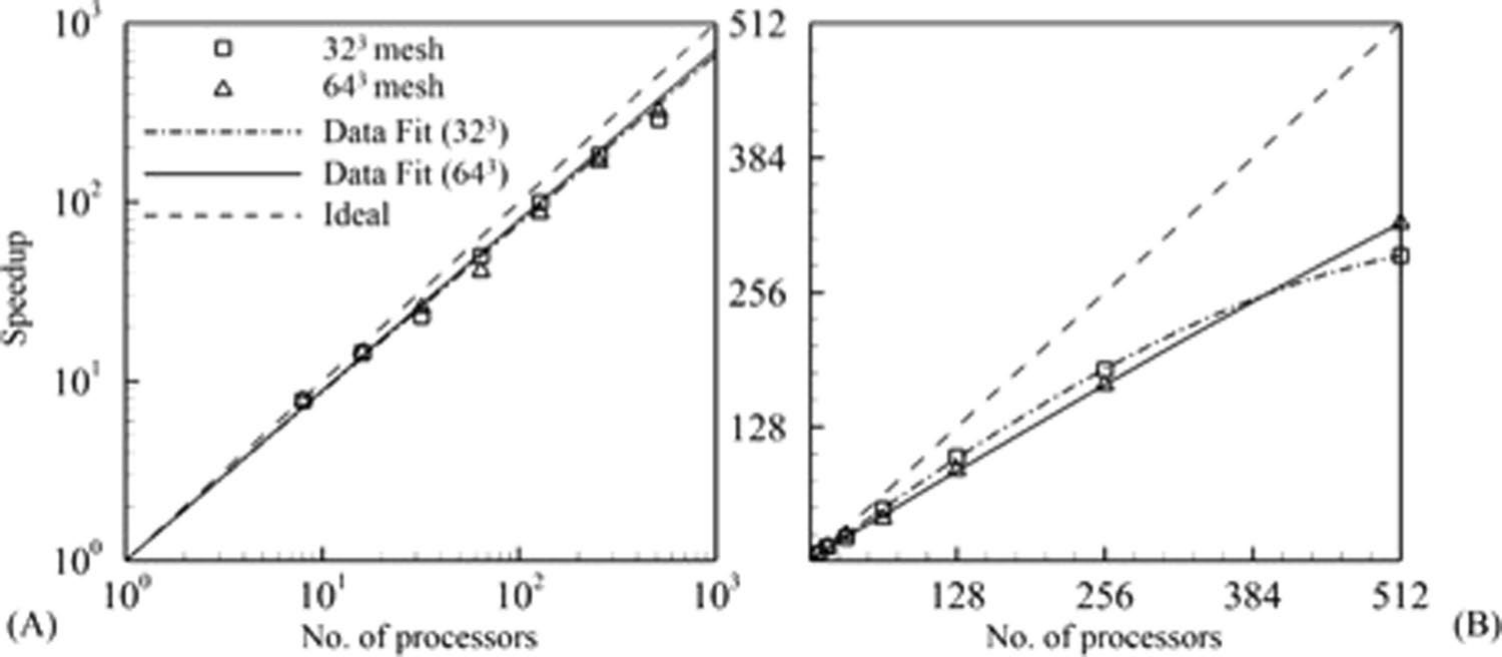
Parallel performance for different number of elements. (**A**) Comparison of speedup on a log-log plot with data fit using power curves and (**B**) speedup on a linear scale plot with data fit using quadratic polynomial.

Figure 8(A) shows that the speedup on a log-log plot is similar for both 32^3^ and 64^3^ cases up to 512 processors. The power data fit to 32^3^ case shows a speedup slope of 0.94 while for 64^3^ it shows 0.95. Based on the data fit the parallel speedup (speedup/ideal) efficiency ranges from 99% for 8 processors to 63% for 512 processors. In Fig. 8(B) the speedup is plotted on a linear scale and the 32^3^ case starts to plateau due to increase in communication time between processors while the 64^3^ case maintains a linear slope. The processors show different performances for different runs since each case is not run on the same server, which gives a deviation in speedup of up to 5%. The initial higher speedup for the 32^3^ case compared to the 64^3^ case is within this tolerance limit. Further improvements can be made by using non – blocking instead of blocking MPI send and receive commands. Also optimizing the domain decomposition can lower communication time.

**Figure 9:**
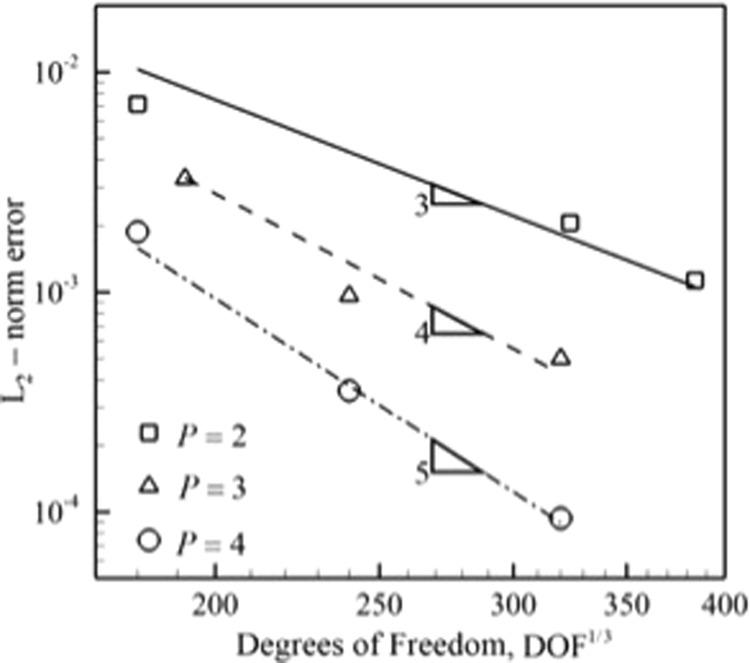
Comparison of rate of convergence for *P* = 2, *P* = 3 and *P* = 4 uniform rectangular elements using the LDG scheme to solve Navier-Stokes equations for isotropic turbulence problem.

The convergence study is shown in Fig. 9 compares the convergence rates for different orders of polynomials. As evident from the plots, the higher order methods show the higher convergence rates in agreement with theory.

A similar scaling study was performed on the Stampede2 machine at the Texas Advanced Computing Center. The study was performed on the new Intel Knights Landing architecture. This new hardware promises much better performance through improved memory bandwidth and larger cache memory per computational core.

**Figure 10:**
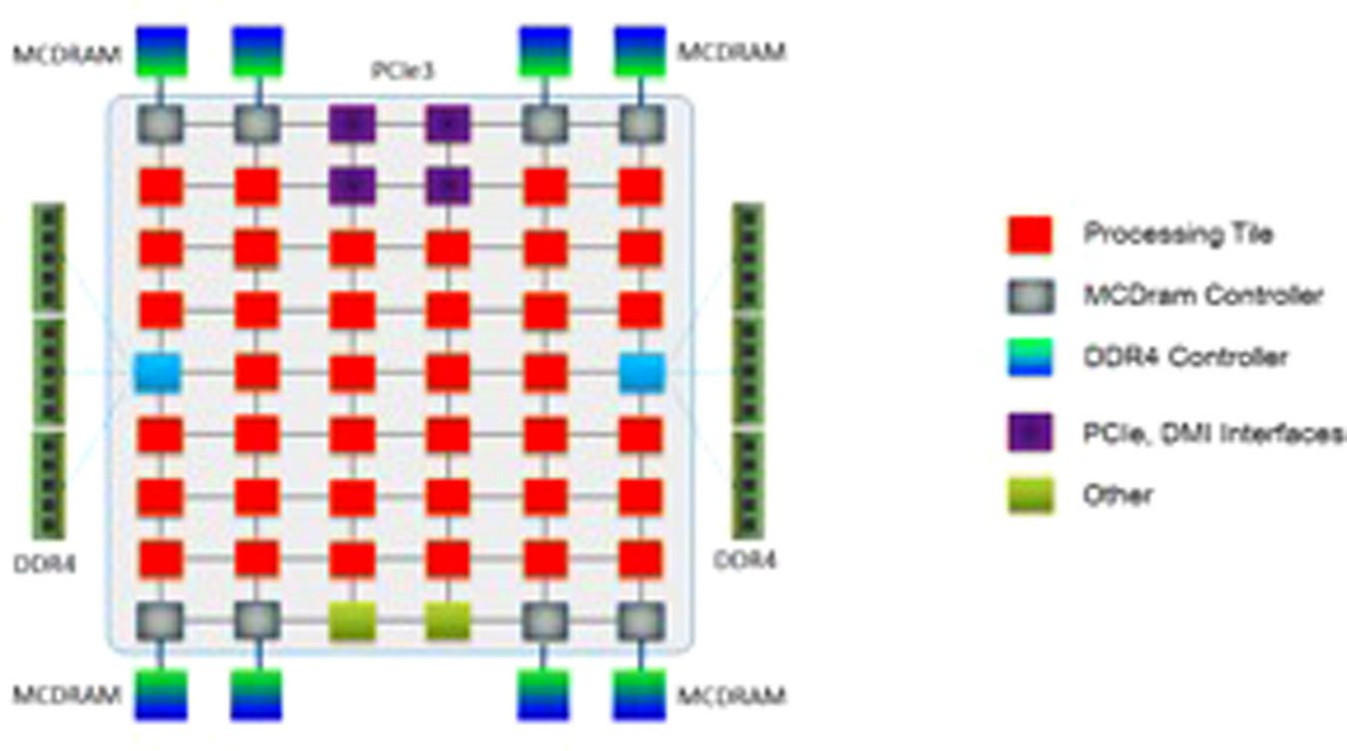
Block Diagram of Intel Knights Landing Processor Architecture (from Intel Website).

The Knights Landing processor from intel consists of 36 active tiles each consisting of 2 processing computational cores, hence having a maximum total of 72 processing cores per compute node (Fig. 10). The cores are connected to each other via a two dimensional on-die ring type interconnect which can deliver an aggregate data bandwidth in excess of 700 gigabytes per second. Each tile containing 2 processing cores shares a 1-megabyte level 2 cache and each compute core has its individual L1 instruction and data caches of 32 kilobytes respectively. Additionally, each core has two vector processing units (VPUs) which allows for very fast floating-point arithmetic operations in parallel.

The local nature of the discontinuous Galerkin method makes it a prime candidate for peak performance on such a vectorized architecture. The fundamental idea behind the Galerkin method is the repeated interpolation and integral computations within each discretized element. As higher orders of the approximation polynomials are employed the amount of computations being performed on the data increases exponentially. This ideally suits the kind of architecture possessed by the Knights Landing processor which relies on bringing in chunks of data from higher level, slow memory like conventional RAM (Random Access Memory) to large caches less frequently and then working on them for longer periods of time.

**Figure 11:**
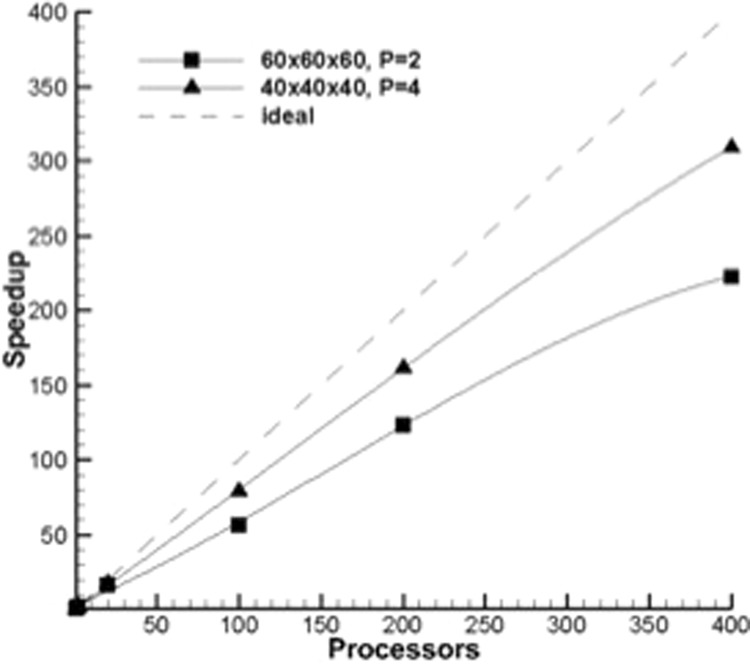
Parallel scaling of MIG code via MPI.

**Figure 12:**
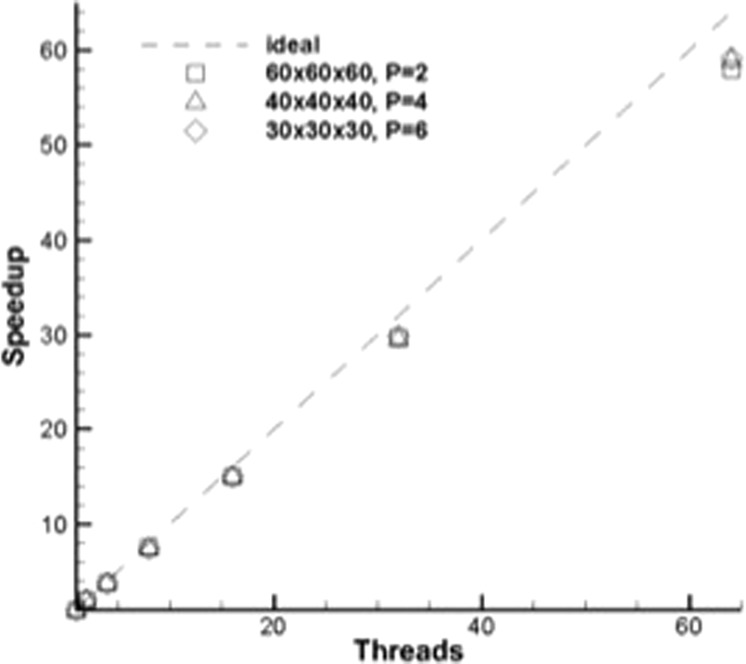
Parallel scaling of MIG code via OpenMP.

Figures 11 and 12 show the scaling of different orders of polynomials with numerical accuracy via MPI and OpenMP implementations of the MIG code. It is quite evident from the initial results that the higher order polynomial solutions show the best promise of parallel scaling. The MPI scaling showed the most dramatic difference between the higher order polynomials. For 100 MPI tasks, the parallel efficiency was 56.6% for *P* = 2, 79.5% for *P* = 4 and 89.0% for *P* = 6. At 400 MPI tasks, the efficiency fell to 55.7% for *P* = 2 and 77.4% for *P* = 4. The OpenMP scaling showed much less of a difference between the three polynomial orders. The code was run for as many as 64 OpenMP threads on the Knights Landing processor. The highest order tested (*P* = 6) was marginally more efficient, with the biggest difference occurring as the number of threads was increased. For 64 threads, the efficiencies were 90.6%, 92.0% and 92.5% for *P* = 2, 4, and 6, respectively. Overall, the OpenMP efficiency was greater than the MPI efficiency for equivalent number of threads and tasks. As described earlier, improvements to the MPI implementation and domain decomposition method can improve the efficiency of the MPI scaling.

For scientific or an engineering problems one is interested in the eventual computational cost for a pre-specified level of acceptable error in the simulations. The combination of a better error convergence rate and superior scaling for higher order methods leads to the evaluation that for reasonably large numerical problem the computational cost for a specific level of numerical error will be the lowest for higher order methods.

## Mach 2.25 Turbulent Boundary Layer Flow

The scheme described in the Numerical Method was used to simulate the zero pressure gradient developing turbulent boundary layer at Mach 2.25. This case serves as a good stepping stone for high speed turbulent flow simulations with higher freestream Mach numbers. The primary challenge in simulating high speed turbulent boundary layers is tied to the large temperature gradient that develops across the boundary layer caused by the increased influence of viscous dissipation (Spina *et al*. 1994).

The freestream conditions and the corresponding range of the momentum thickness-based Reynolds number ${{\rm{Re}}}_{\theta }={u}_{\infty }\theta /{\nu }_{\infty }$ (based on the freestream viscosity as opposed to the wall viscosity) for the present case are listed in Table 5. In order to transition the flow to turbulence, the flow is tripped using the method of Schlatter and Örlü (2012) through bypass transition. The tripping body force is in the wall-normal direction, and it is given by

40 $${f}_{y}=\exp [{[(x-{x}_{0})/{\ell }_{x}]}^{2}-{[y/{\ell }_{y}]}^{2}]g(z,t)$$

**Table 5:** Freestream conditions for the turbulent boundary layer flow.

Parameter	Value
*u*_∞_	745.2 m/s
*p*_∞_	10.13 kPa
*T*_∞_	273 K
*T*_∞_	2.25
Re_∞_	400–1350

The forcing function g(z,t) in Eq. (12) fluctuates as a function of time, and it also contains a random coefficient which varies in the spanwise direction. The full form of the forcing function is given in Schlatter and Örlü (2012).

The computational domain consists of ${N}_{x}\times {N}_{y}\times {N}_{z}=900\times 64\times 64$ finite elements. Within each element, a modal basis function representation is employed. Quadratic Legendre basis functions (p = 2) with third-order spatial accuracy are used to interpolate the solution. The grid in the direction normal to the wall is stretched, with the smallest grid spacing (based on the inner wall units) of Δy^+^ = 0.5 at the wall. In the spanwise and streamwise directions, the grid is uniform. The boundary conditions for the computational domain are as follows. The laminar boundary layer solution is used as an inflow boundary condition for the simulation. At the wall, the adiabatic and no-slip boundary conditions are prescribed. In the spanwise direction, symmetric boundary conditions are enforced. In the streamwise direction, a fringe region is added at the outlet to eliminate reflections from the outlet boundary. This concept has been successfully used in simulations of turbulent boundary layers in the past, *e.g*. Spalart and Watmuff^42^.

### Instantaneous flow field

**Figure 13:**
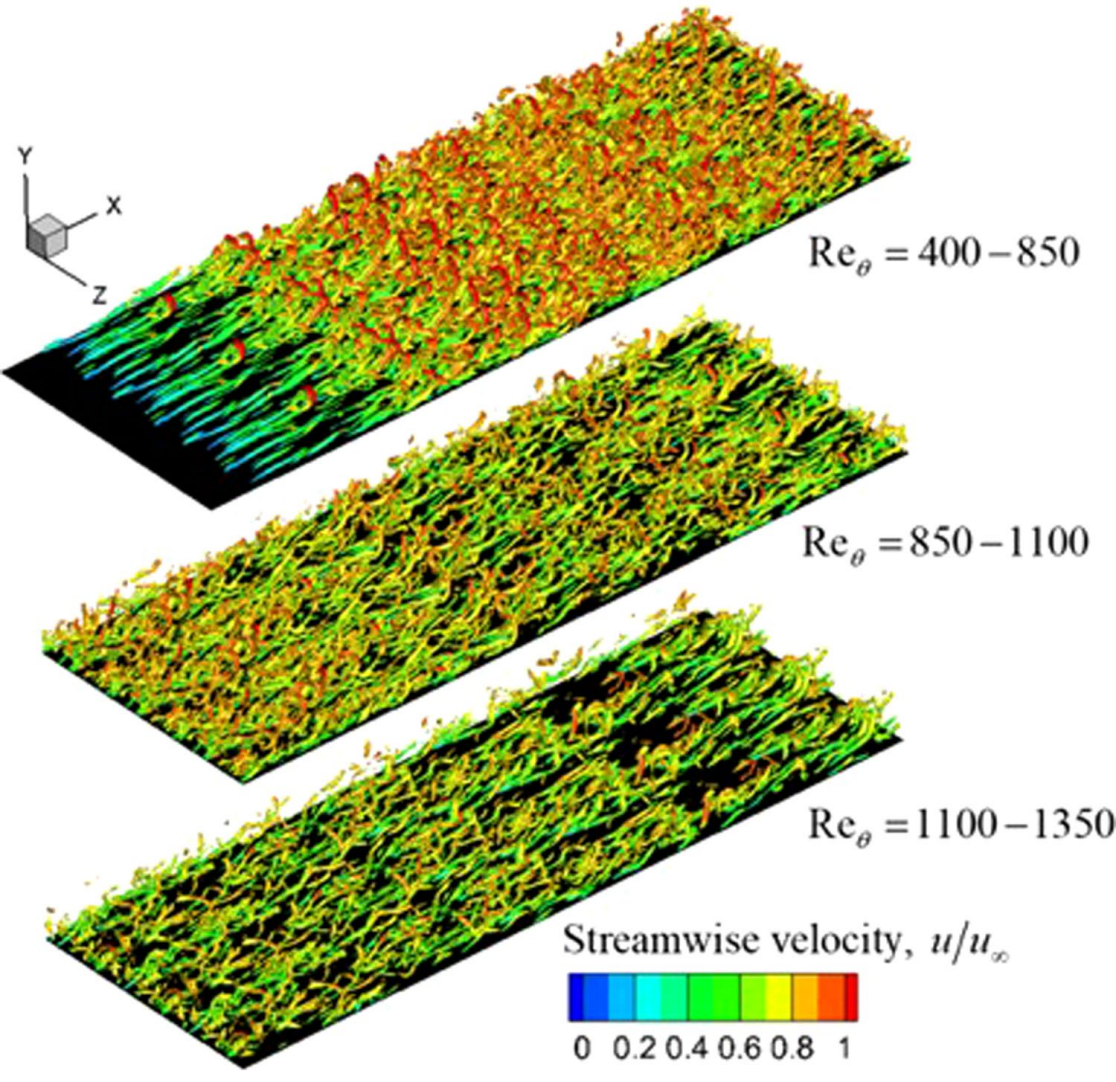
Iso-surfaces of the instantaneous normalized Q criterion (Q = 3). The domain is duplicated three times for visualization purposes.

The features of the instantaneous flow are studied in Fig. 13, which gives the plot of the Q criterion iso-surfaces at the value of Q = 3. The iso-surfaces are colored by the magnitude of the streamwise velocity for a momentum thickness Reynolds number Re_*θ*_ in the range of 400-1350. The plane below the iso-surfaces represents the flat plate. The flow is tripped and the initial coherent structures quickly break down into a fully turbulent flow. Asymmetric one-legged hairpin vortices can be observed along with the more typical structures. The flow in Fig. 13 is plotted after both the initial transients disappeared and the mean flow calculations were carried out, corresponding roughly to three flow-through times (the fluid convecting three times over the streamwise length of the plate).

**Figure 14:**
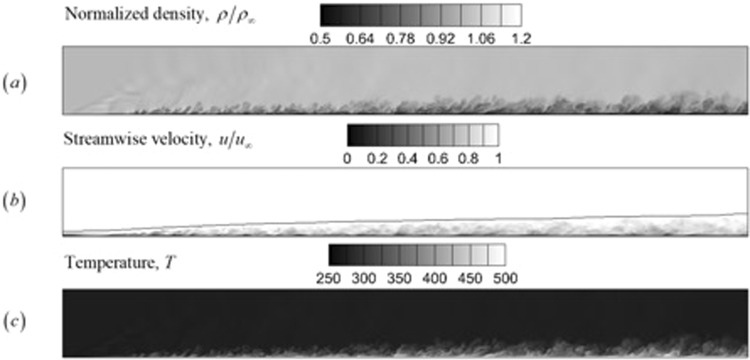
Instantaneous contours for the turbulent boundary layer at M = 2.25. (**a**) Normalized density field, (**b**) normalized instantenous velocity, and (**c**) temperature (K) are plotted.

Figure 14 shows the instantaneous normalized density, normalized streamwise velocity and temperature fields for the flow after two flow-through times. The slice location of the xy-plane corresponds to the center of the domain in the spanwise direction (z = 1.5 mm and k = 32). The height of the domain in the wall normal direction was found to be sufficient to avoid the reflections from the top boundary destroying the rest of the solution. A large-scale structure angle of about 45 degrees has been observed for this problem, along with a shallower 10 degree angle for structures closer to the wall. These angles can also roughly be seen in Fig. 14, despite the lesser level of resolution in this study in comparison with that of Poggie^43^.

The ratio of the freestream temperature to the wall temperature matches the expected value that can be obtained from a similarity solution of a compressible laminar boundary layer with the adiabatic wall boundary condition. The growth of the boundary layer is shown in Fig. 14(b) by plotting the boundary layer thickness.

**Figure 15:**
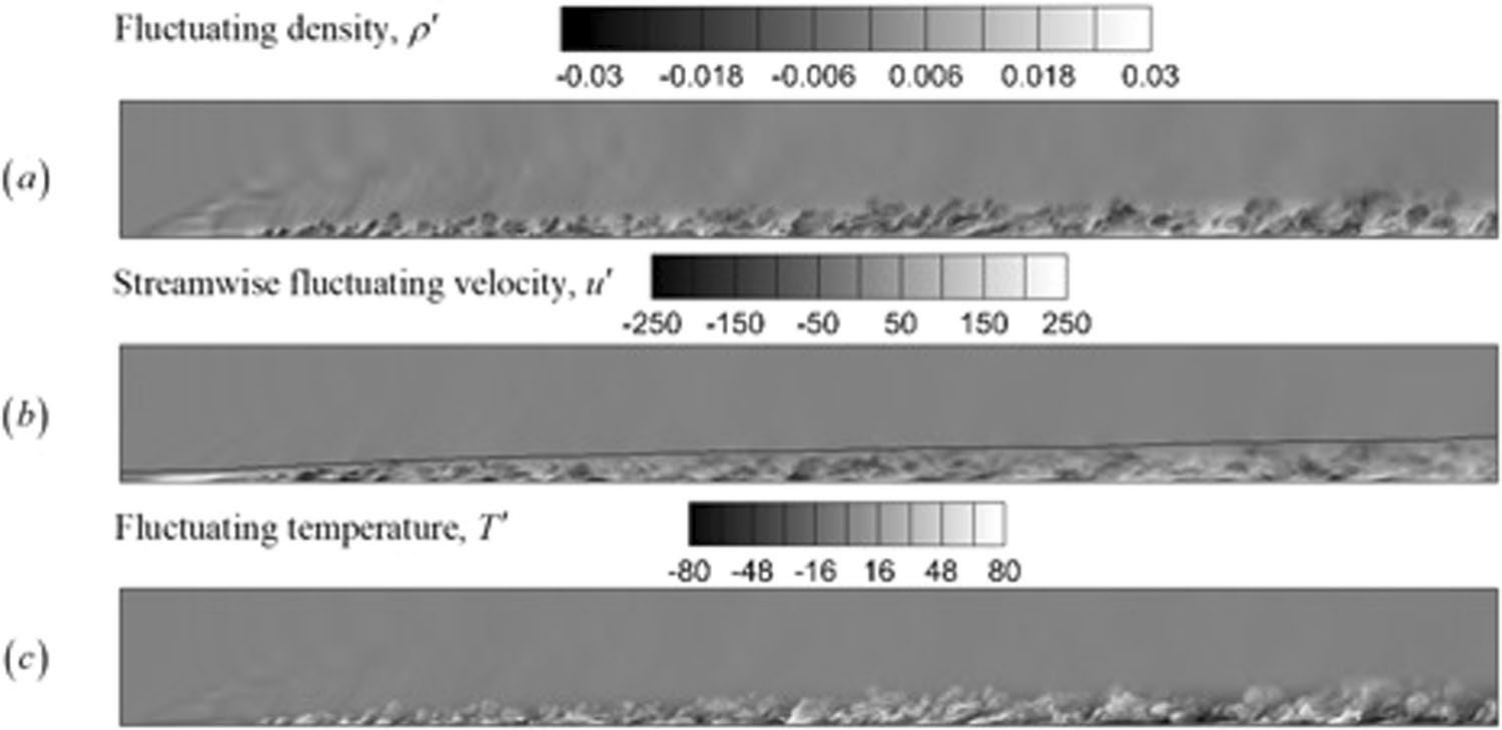
Instantaneous contours for the turbulent boundary layer at M = 2.25. Fluctuations of the (**a**) normalized density field, (**b**) normalized instantenous streamwise velocity, and (**c**) temperature (K) are plotted.

Figure 15 shows the fluctuations of the normalized density, normalized streamwise velocity and temperature fields. The xy-plane slice location is identical to that in Fig. 14. The fluctuations are plotted to offer additional visualization of the turbulent flow field. The high intensity fluctuations in the boundary layer show packets of fluid which are hotter and lighter than the freestream fluid and which are pushed upward as the boundary layer grows.

**Figure 16:**
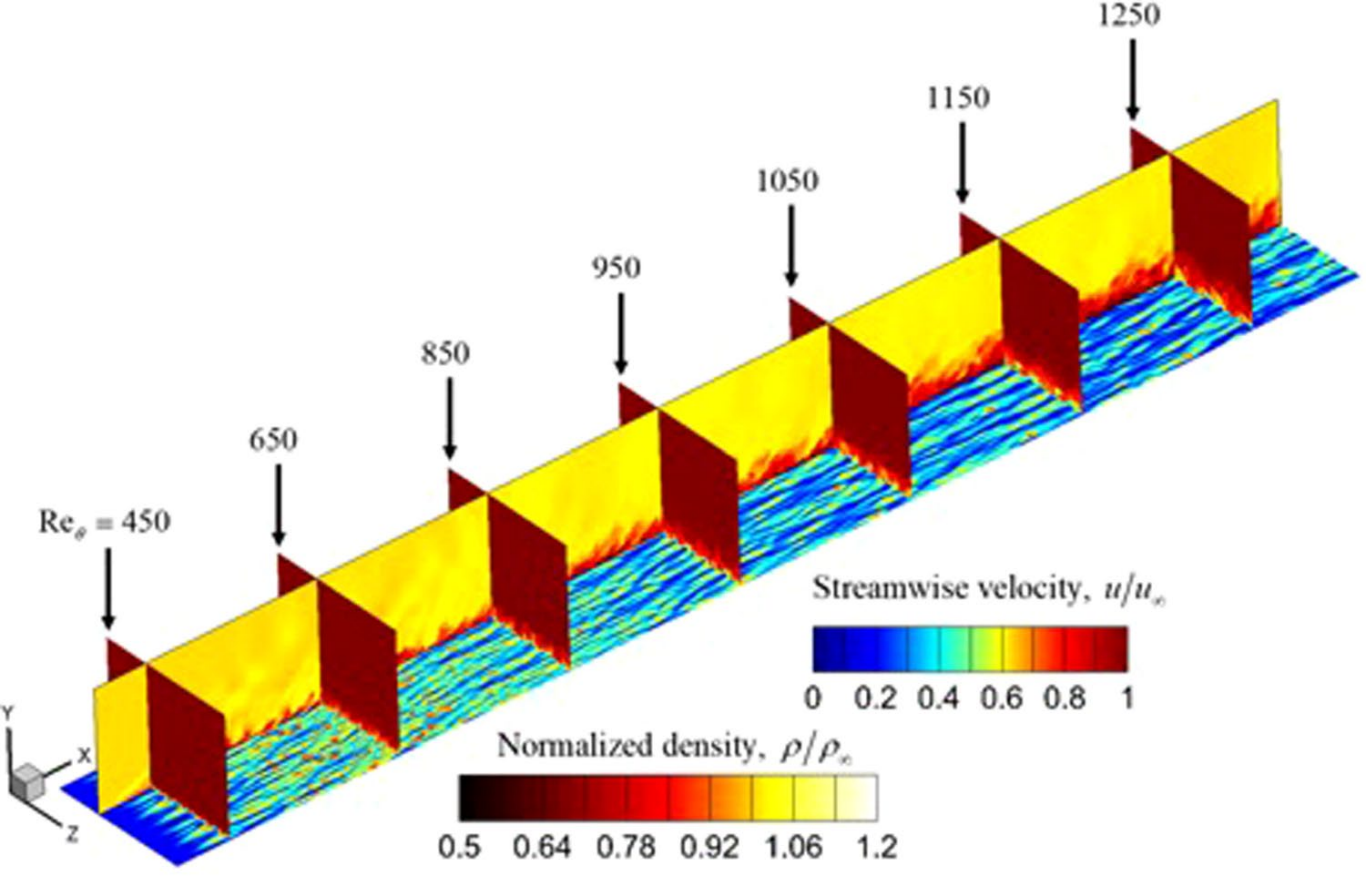
Streamwise velocity plotted at the wall-normal direction plane corresponding to y ≈ 6 × 10^−5^ (j = 15) and several planes along the streamwise direction. Normalized density is plotted for a spanwise plane to illustrate the boundary layer growth and the flow structures.

In Fig. 16, the flow structures along a wall normal plane are visualized by plotting the velocity contours at the j = 15 plane (y ≈ 6 × 10^−2^ mm). The vortices are seen to produce low speed streaks which spread out as the flow becomes fully turbulent. The figure also shows that the fluid in the boundary layer is drawn upward away from the wall. The normalized density profile is plotted along various spanwise planes and shows the growth of the flow structures as the thickness of the boundary layer increases.

### Mean Flow Field

**Figure 17:**
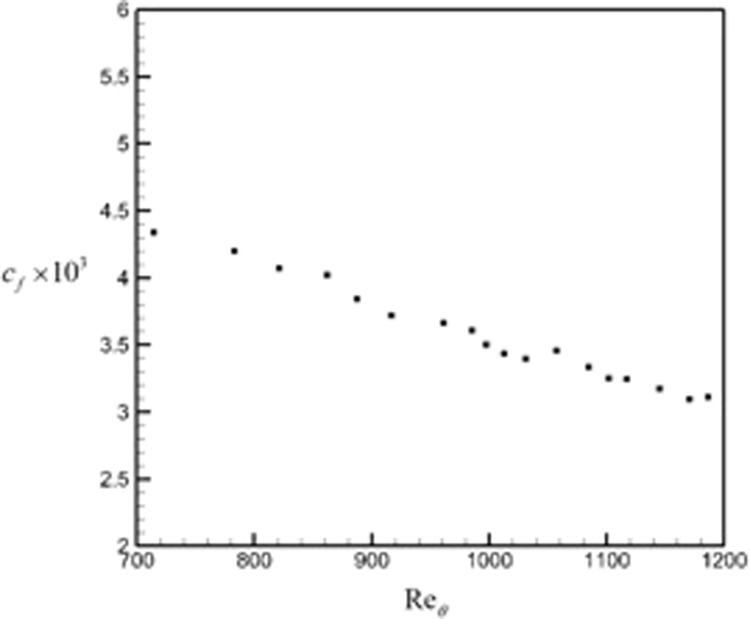
Skin friction coefficient plotted versus the momentum thickness-based Reynolds number in the range 700–1200.

Figure 17 shows the skin friction coefficient plotted against the momentum thickness Reynolds number in the range of Re_*θ*_ = 700–1200. This range is chosen because the flow has already become fully turbulent. The skin friction decreases with increasing momentum thickness Reynolds number.

**Figure 18:**
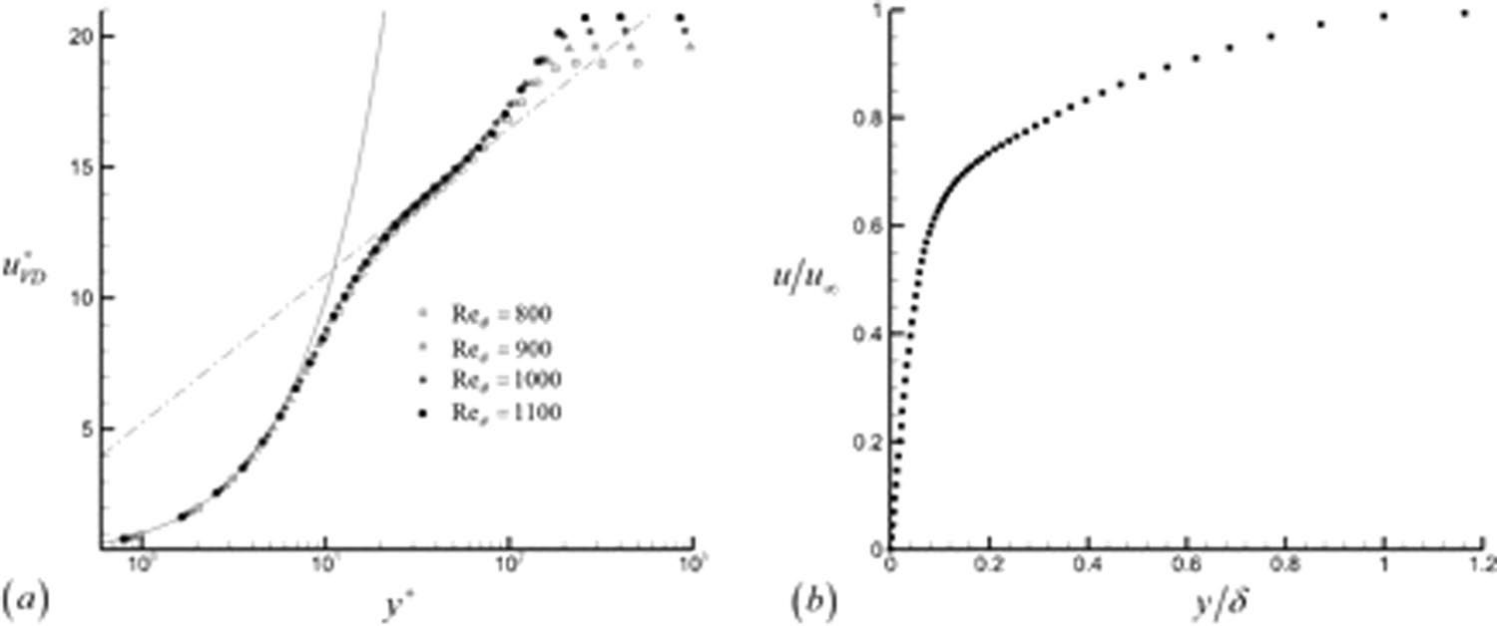
(**a**) The van Driest transformed velocity plotted in the inner wall coordinates for multiple downstream locations, and (**b**) the mean streamwise velocity plotted in the outer coordinatesat the downstream location corresponding to Re_θ_ = 1000.

Figure 18 plots the mean velocity profiles for the turbulent boundary layer, scaled in both the inner and outer coordinates. For the inner wall coordinates, the mean velocity is plotted in the van Driest-transformed form^26^. The transformed velocity is given by

41 $${u}_{VD}^{+}={\int }_{0}^{{u}^{+}}{(\frac{\bar{\rho }}{{\bar{\rho }}_{w}})}^{1/2}d{u}^{+}$$

The transformed velocity is plotted at downstream locations of Re_*θ*_ = 800–1100. The streamwise velocity profiles are seen to collapse reasonably well to the law of the wall in the viscous sublayer (indicated by a solid line in Fig. 18) and the buffer layer (dashed and dotted line in Fig. 18) for this particular case.

**Figure 19:**
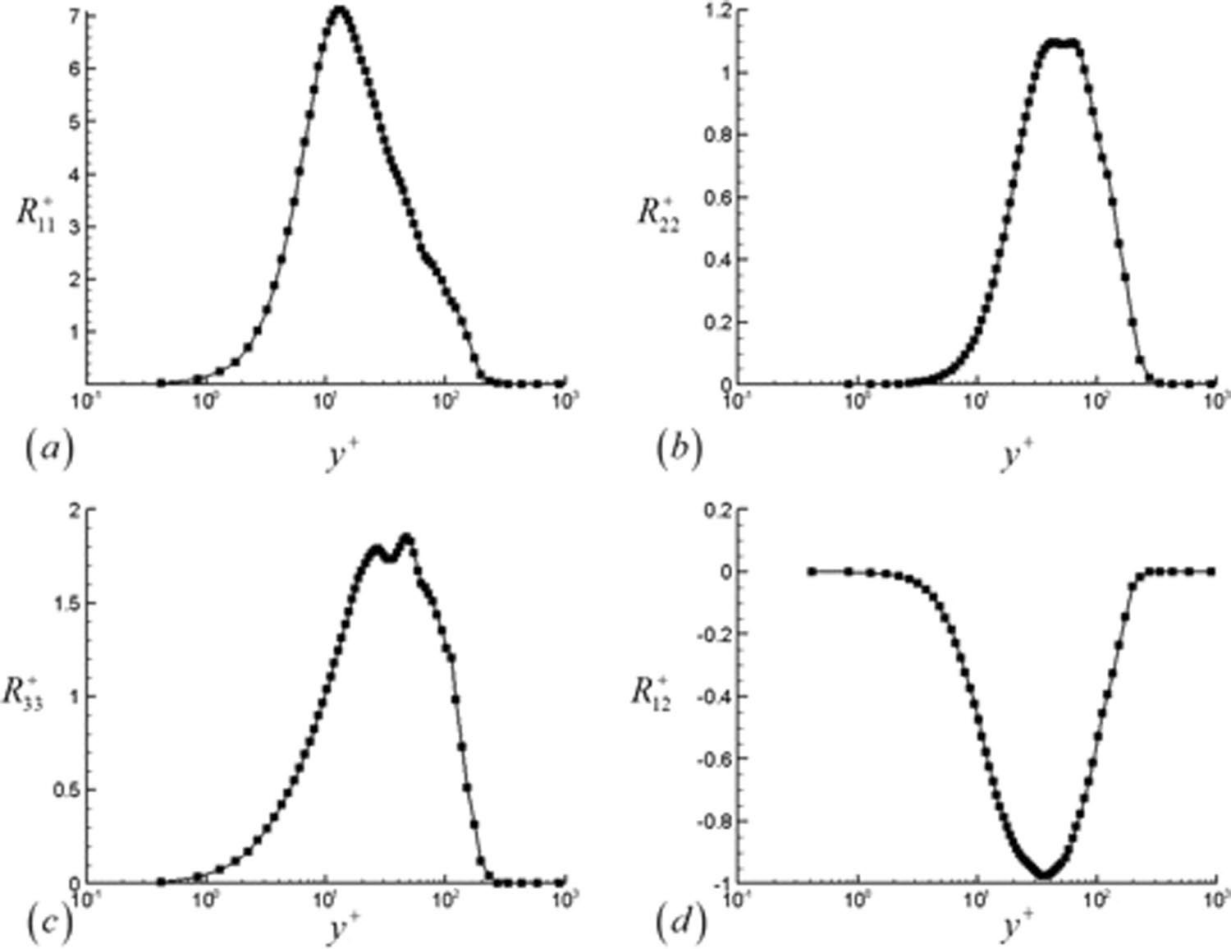
Transformed Reynolds stresses plotted in the inner wall coordinates at the downstream location corresponding to Re_θ_ = 1000. (**a**) Streamwise, (**b**) wall normal, (**c**) spanwise and (**d**) u′v′ components are shown.

**Figure 20:**
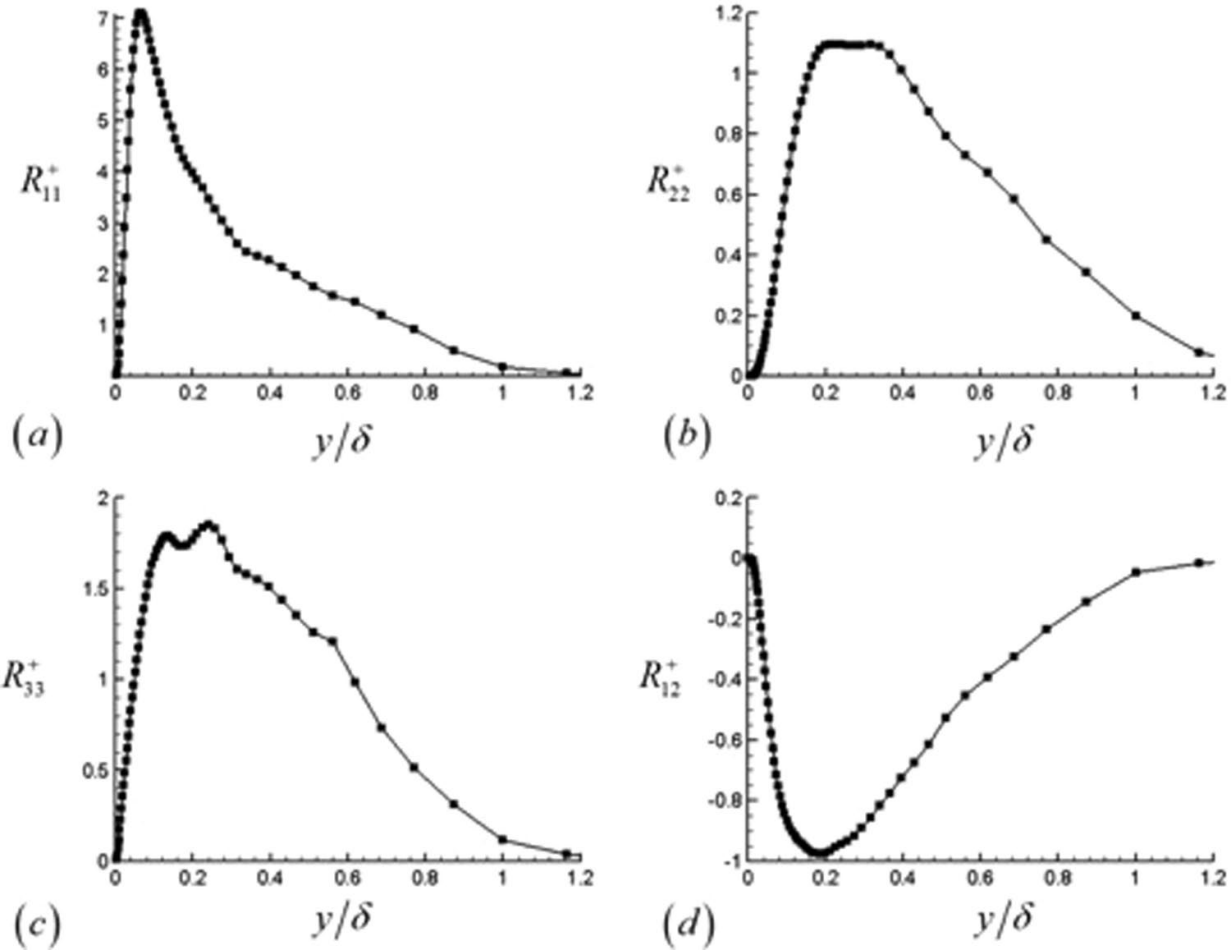
Transformed Reynolds stresses plotted in the outer coordinates at the downstream location corresponding to Re_θ_ = 1000. (**a**) Streamwise, (**b**) wall normal, (**c**) spanwise and (**d**) u′v′ components are shown.

Figures 19 and 20 give the Reynolds stresses scaled by the wall shear stress. The transformed Reynolds stress is plotted using the inner coordinates in Fig. 19 and the outer coordinates in Fig. 20. The transformed Reynolds stress is calculated as

42 $${R}_{ij}^{+}=\frac{\bar{\rho }\overline{{u}_{i}^{^{\prime} }{u}_{j}^{^{\prime} }}}{{\tau }_{w}}=\frac{\bar{\rho }\overline{{u}_{i}^{^{\prime} }{u}_{j}^{^{\prime} }}}{{\bar{\rho }}_{w}{u}_{\tau }^{2}}$$

The Reynolds stresses are plotted at the downstream location of Re_*θ*_ = 1000, which is not in close proximity to the outflow boundary but still sufficiently far from the flow tripping region. At this location, the peak value of the normal Reynolds stress occurs at approximately y^+^ = 11 (y/δ = 0.06). The wall normal and spanwise components of the Reynolds stress are smaller in comparison, and their peak values occur further away from the wall. After reaching their peak values, all the stresses decay with increasing y/δ sufficiently far away from the wall. The Reynolds stresses indicate that the majority of the turbulence is produced in the region of y^+^ = 10–100, fairly close to the wall.

## Conclusions

A scalable, parallel, high-fidelity DG formulation was demonstrated for the test case of isotropic turbulent flow for a Taylor-Green vortex problem for Reynolds numbers ranging between 100–1600. The solutions for the DG ILES method were found to match up well with DNS results up to the Reynolds number of 1600, for which a higher resolution is necessary. Tests using the Godunov and LLF numerical fluxes showed negligible differences in the dissipation rates for polynomial orders beyond *P* = 2. The kinetic energy dissipation rate was found to converge to the DNS solution when a higher number of DOFs was used.

A Reynolds number of 1600 was then used to study the performance of different polynomial orders from *P* = 2 to *P* = 6 with regard to computational cost and scalability in parallel. The DG method demonstrated the advantages of the higher-order polynomials for parallel implementation. Namely, the higher-order polynomials showcased superior scalability and performance to achieve a given level of error over the range that was tested. The studies showed that increasing the order of the interpolating polynomial increased the parallel efficiency using both the MPI and OpenMP parallel implementations. The improvement in parallel efficiency was larger for the MPI implementation than for the OpenMP implementation. The MPI implementation showed improvements of 20–30% in parallel efficiency between *P* = 2 and *P* = 6 depending on the number of tasks. The difference between *P* = 2 and *P* = 6 for the OpenMP implementation was as small as 2%. It is not conclusive from this study whether this behaviour continues into higher order polynomials or if the efficiency saturates.

The same parallel framework was used to compute simulations of the development of a supersonic turbulent boundary layer at Mach 2.25. These computations employed nearly 33 million spatial degrees of freedom with the solution domain being approximated with quadratic Legendre polynomials. Numerical investigations into the physics such as the variation of the skin friction coefficient with the Reynolds number and characteristics of the Reynolds stress in the boundary layer are presented. These computations provide confidence in the capabilities of the numerical framework to perform more investigations to provide deeper physical insights in such phenomena in future work. The parallel scalability and efficiency of this framework suggests that such large studies can now be attainable in reasonable amount of time.

The focus of future studies is exploring polynomial orders beyond *P* = 6 to determine the diminishing returns on scaling when continuing to increase the polynomial order. In addition, domain decomposition will be performed by minimizing the surface area over which separate MPI tasks are required to communicate with each other. These changes should improve the scaling of the MPI implementation, which is not as optimized as the OpenMP scaling at the present time.

## Data Availability

The data for the presented results is available and will be posted on a public link at APRG website.
