# Author Correction: Supersonic turbulent flow simulation using a scalable parallel modal discontinuous Galerkin numerical method

**DOI:** 10.1038/s41598-020-58144-x

**Published:** 2020-01-21

**Authors:** Tomas Houba, Arnob Dasgupta, Shivasubramanian Gopalakrishnan, Ryan Gosse, Subrata Roy

**Affiliations:** 1 SurfPlasma Inc, Gainesville, FL 32601 USA; 2 Applied Physics Research Group, Department of Mechanical and Aerospace Engineering, University of Florida, Gainesville, FL 32601 USA; 3 Department of Mechanical Engineering, Indian Institute of Technology Bombay, Mumbai, 400076 India; 4 Vanilla Peak Ct, Vail, AZ 85641 USA

Correction to: *Scientific Reports* 10.1038/s41598-019-50546-w, published online 08 October 2019

Two references cited in the body of the Article as Spina *et al*. 1994 and Schlatter & Örlü 2012 (section Mach 2.25 Turbulent Boundary Layer Flow) were omitted from the References section.

The papers are listed below as References 1 and 2.

Spina, E. F., Smits, A. J. & Robinson, S. K. The physics of supersonic turbulent boundary layers. *Annual Review of Fluid Mechanics* **26**(1), 287–319 (1994).

Schlatter, P. & Örlü, R. Turbulent boundary layers at moderate Reynolds numbers: inflow length and tripping effects. *Journal of Fluid Mechanics* **710**, 5–34 (2012).

